# Phytochemical profiling, antimicrobial, antibiofilm, and molecular docking of *Farsetia aegyptia* and *Zilla spinosa* from Saudi Arabia

**DOI:** 10.3389/fphar.2025.1723207

**Published:** 2026-02-03

**Authors:** Malek Besbes, Assia Hamdi, Mabrouk Horchani, Kaouther Majouli, Amal Dbeibia, Saoussen Jilani, Abeer Ayed Alshammari, Salwa Ahmed Lotfi, Khulood Fahad Alabbosh, Ramzi Hadj Lajimi, Hichem Ben Jannet, Jamil Kraiem, Walid Ben Selma

**Affiliations:** 1 Department of Biology, College of Sciences, University of Hail, Ha’il, Saudi Arabia; 2 Laboratory of Chemical, Pharmaceutical and Pharmacological Development of Drugs, Faculty of Pharmacy, University of Monastir, Monastir, Tunisia; 3 Laboratory of Heterocyclic Chemistry, Natural Products and Reactivity (LR11Es39), Medicinal Chemistry and Natural Products, Faculty of Science of Monastir, University of Monastir, Avenue of Environment, Monastir, Tunisia; 4 College of Science and Humanities-Al-Duwadmi, Shaqra University, Riyadh, Saudi Arabia; 5 Laboratory of Analysis, Treatment and Valorization of Environmental Pollutants and Products, Faculty of Pharmacy, Monastir University, Monastir, Tunisia; 6 Department of Chemistry, College of Science, University of Ha’il, Ha’il, Saudi Arabia

**Keywords:** antibiofilm effects, antimicrobial activities, *Farsetia aegyptia*, molecular docking, phytochemical analysis, *Zilla spinosa*

## Abstract

**Background:**

*Farsetia aegyptia* and *Zilla spinosa* belonging to the Brassicaceae family, well-known for their biological and therapeutic activities.

**Objective:**

To our knowledge, this is the first comprehensive study to explore the composition, antibacterial, anticandidal, antibiofilm activities using *in vitro*, and molecular docking analysis assays of *F. aegyptia* and *Z. spinosa* herb extracts collected from the Hail region of Saudi Arabia.

**Methods:**

The antibacterial and anticandida effects of chloroform, ethanolic, and aqueous extracts from the aerial parts of *F. aegyptia* and *Z. spinosa* were determined by conventional assays. The compositions of extracts were determined by Gas Chromatography–Mass Spectrometry (GC–MS) and High-Resolution Liquid Chromatography–Mass Spectrometry (HR–LC–MS). Molecular docking simulations were carried out with the major identified compounds against penicillin-binding protein 4.

**Results:**

The chloroform and ethanolic extracts of these plants exhibited substantial activities against *Enterococcus faecalis* ATCC 29212, and *Listeria monocytogenes* ATCC 19115, with minimum inhibitory concentration (MIC), minimal bactericidal concentration (MBC) levels varying between 625 and 2,500 μg/mL, and MBC/MIC was equal to 1. The chloroformic extract of *F. aegyptia* demonstrated the most significant anticandidal activity against *Candida albicans* ATCC 90028 and *Candida krusei* ATCC 6258, with MIC of 625 μg/mL. Interestingly, the chloroform extract of *F. aegyptia* demonstrated the most important antibiofilm activity against *Staphylococcus aureus*, with Minimum Biofilm Inhibition Concentration (MBIC50) of 700 μg/mL, while the chloroform extract of *Z. spinosa* showed the most potent antibiofilm activity against *Pseudomonas aeruginosa* ATCC 27853 with MBIC50 = 630 μg/mL. *Candida albicans* showed the highest sensitivity to the ethanolic extract of *Z. spinosa*, with MBIC50 = 660 μg/mL. The GC–MS analysis identified β-sitosterol (40.39%), stigmasterol (22.24%), and coumarin (9.25%), as the main components of *Z. spinosa*; and linolenic acid (12.68%), linolenic acid ethyl ester (7.31%), arachidonic acid (6.96%), and (Z)-13-docosenamide (6.47%) as predominant compounds in *F. aegyptia*. The docking analysis revealed that the key compound stigmasterol from *Z. spinosa* served as superior ligands, penicillin-binding protein four (PBP4) (−7.7 kcal/mol).

**Conclusion:**

These findings highlight the powerful antimicrobial and antibiofilm activities of *F. aegyptia* and *Z. spinosa*, and supporting their prospective role in developing novel anti-infective agents.

## 1 Introduction

One of the world’s greatest challenges is the rise, the rapid and widespread spread of antimicrobial resistance in pathogenic Gram-negative and Gram-positive bacterial strains, which were associated with multi-drug resistant (MDR) bacterial infections responsible for millions of deaths each year for several important reasons: current medications are often ineffective against them, the scarcity of new antibiotics, and synthetic antibiotics can have harmful side effects (WHO, 2024; Khameneh et al., 2019).

Pathogenic microorganisms can form biofilms, which are structured communities encased in an extracellular matrix of polymeric materials (Coppola et al., 2025; Kungwani et al., 2024; Alibi et al., 2021). These biofilms can be highly detrimental and are a significant cause of nosocomial infections (Kungwani et al., 2024; Alibi et al., 2021). The National Institutes of Health reported that 80% of chronic infections and 65% of microbial infections are associated with biofilm formation.

To address these challenges, there is a critical need for new, effective drugs to treat pathogenic and MDR bacterial infections. In this setting, the World Health Organization (WHO) has announced an urgent need to encourage the research and development of new drugs or strategies (WHO, 2024). Thus, numerous studies have been conducted to discover novel antimicrobials for clinical use for the eradication of MDR bacteria. However, as no new antimicrobial has been found to combat MDR Gram-negative and Gram-positive bacteria, other strategies are proposed and evaluated *in vitro*. One of these approaches is based on investigating natural products due to their great diversity and relative abundance. Hence, natural products, such as essential oils, which comprise a wide range of chemical compounds, are becoming an attractive and popular mainstream platform for researchers in drug discovery. Therefore, our studies and others have previously reported interesting effects of different plant essential oils extracted from medicinal plants such *Thymus capitatus* (Ben Selma et al., 2024a), *Thymus algeriensis* (Jilani et al., 2025), *Thymus vulgaris* (Ben Selma et al., 2024a; Alibi et al., 2020), Grantia aucheri (Sharifi-Rad et al., 2022a), *Cinnamomum zeylanicum* (Ben Selma et al., 2024b; Alibi et al., 2022), *Teucrium polium* (L.) (Sharifi-Rad et al., 2022b), *Astragalus* species (Zengin et al., 2022) against MDR and extensively resistant Gram-negative and Gram-positive pathogenic bacteria.

Accordingly, secondary metabolites derived from medicinal plants have shown promising effects in combating these infections and are generally considered safe due to their minimal side effects (Cowan, 1999; Gyawali and Ibrahim, 2014; Narayana et al., 2017). This growing interest in plant-based compounds has led to increased engagement from the pharmaceutical industry (Anand et al., 2020). With over 350,000 plant species on Earth (Borris, 1996), many plant-derived products are already being used as commercial antimicrobial drugs (Khameneh et al., 2019).

The Brassicaceae family, a group of dicotyledonous angiosperms (Chen et al., 2011), contains 3,709 species, across 338 genera (Al-Shehbaz et al., 2006). This family is of significant nutritional and economic value and has been a focus of research for many years. The species *Farsetia aegyptia* and *Zilla spinosa*, selected in this study, both belong to the Brassicaceae family.

*Zilla Spinosa* is also an important source of phytoconstituents, such as flavonoids, sterols, glycosides, tannins, triterpenoids, carbohydrates, and glucosinolates (Karawya 1974). These components confer it powerful antiviral, hepatoprotective, antifungal and antioxidant (El-Sharkawy et al., 2013; El-Toumy et al., 2011), hepatoprotective, important anti-inflammatory, antipyretic, and analgesic prospective effects and anti-cancer potential (Ullah et al., 2020; El-Sharabasy and Mohamed, 2013 Suleiman and Ateeg, 2020; Alghanem et al., 2018; Salma et al., 2018; Ullah et al., 2018) of the different fractions of this plant. Additionnally, it has been frequently employed in folk medicine to treat a variety of ailments, including diabetes, gastrointestinal issues (Sekkoum et al., 2015), urinary tract pain (Sekkoum et al., 2011), kidney stones, gallbladder problems, pancreatic and hepatic pain, diarrhea, and respiratory infections (Berreghioua et al., 2014).

*Farsetia aegyptia* organic extracts are rich in bioactive phytocompounds including phenolic acids, glycosinolates, and flavonoids such as kaempferol-7, 8-dilucoside and apienin; flavanols and thier glycosides such as botulin, friedelin, β-amyrin, scopoletin and coumarin (El-Sharkawy et al., 2013). These compounds exhibited different promising biological activities including antimicrobial, antioxidative (Suleiman, and Ateeg, 2020; Atta et al., 2013), anti-inflammatory (Mecheri et al., 2023) hepatoprotective, anticancer and cytotoxic effects against certain selected cancerous cells (Madouh, and Davidson, 2024; Marzouk 2009). Moreover, this plant has been used in traditional medicine for its antispasmodic and anti-diabetic properties and is also utilized to treat rheumatic pains (Youssef, 2013). Moreover, a decoction of this plant can be used for mouth disinfection (Abdelshafeek et al., 2016; Bhandari et al., 2015).

A literature survey indicated that no data are available on the chemical composition and antimicrobial effects of the two plants *F. aegyptia* and *Z. spinosa* growing in Hail region of Saudi Arabia. Thus, the current study aimed to determinate the phytochemical composition of most active herb extracts of these two plants by GC-MS and HR–LC–MS, as well as, to explore their extracts’ eventual antibacterial, anticandidal, antibiofilm activities, and to conduct a docking analysis to predict molecular interactions between major compounds of active extracts and penicillin-binding protein 4 (PBP4) of *Enterococcus faecalis* (PDB: 6mki) implicated in the pathway of biosynthesis of peptidoglycans.

## 2 Methods

### 2.1 Reagents and chemicals

All solvents used in this study—ethanol (99.99%, C2H6O), chloroform (90%, CHCl3), and dimethyl sulfoxide (DMSO)—as well as the Mueller–Hinton broth, resazurin, filter paper, Amphotericin B, and ciprofloxacin employed in the experiments—were purchased from Merck (Darmstadt, Germany).

### 2.2 Preparation of plants extract

**FIGURE 1:**
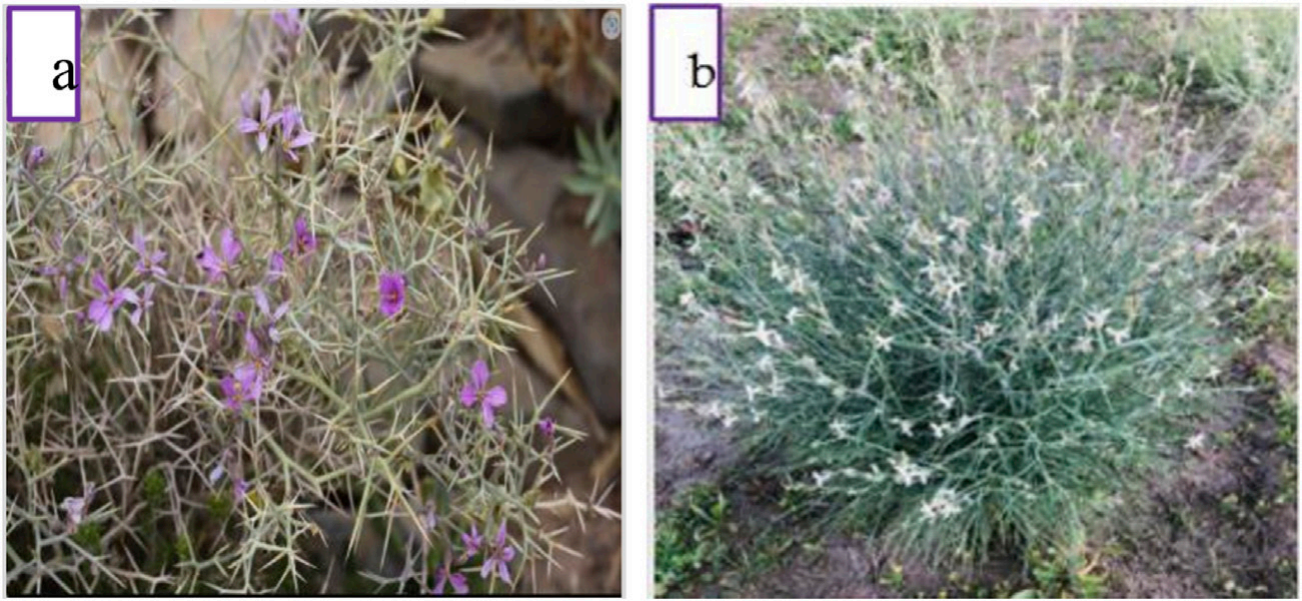
**(a)** Zilla spinosa (L): Prantl (Z.s) **(b)** Farsetia aegyptia Turra (F.a).

*Farsetia aegyptia* and *Z. spinosa* (Figure 1) were collected from farms in the Simira governorate, located south of Hail, Saudi Arabia, in 2022, after receiving verbal permission from the owners. These plants were taxonomically identified by Dr. Assia Hamdi at the Faculty of Pharmacy, Monastir University, Tunisia. Voucher specimens were prepared for each species and deposited at the herbarium of the Laboratory of Biology (HLB) at the College of Sciences, University of Hail, Saudi Arabia, as follow: Plant 1: *F. aegyptia* Turra Voucher No. F.a 1, and Plant 2: *Z. spinosa* L. Voucher No. Z.s 1.

These voucher specimens confirm the botanical identities and serve as references for future use.

Following the extraction protocol established by our research group (Besbes et al., 2025), the preparation of extracts was performed as follows: after drying and grinding approximately 700 g of plant material, the maceration method was used to extract compounds in chloroform, ethanol, and water. The extraction yields were 1.4% and 2.1% (chloroform), 5.17% and 6.21% (ethanol), and 7.2% and 8.6% (water) for *F. aegyptia* and *Z. spinosa*, respectively. All samples were stored in airtight glass vials sealed with aluminum foil at −4 °C until further use.

### 2.3 Microorganisms strains

Three Gram-positive bacterial strains including: *Staphylococcus aureus* ATCC 25923, *Listeria monocytogenes* ATCC 19115, and *E. faecalis* ATCC 29212; three Gram-negative bacteria strains comprising: *Escherichia coli* ATCC 35218, *Salmonella* Typhimurium ATCC 1408, and *Pseudomonas aeruginosa* ATCC 27853; and three strains of *candida*: *Candida neoformans* ATCC 14116, *Candida albicans* ATCC 90028, and *Candida krusei* ATCC 6258.

### 2.4 Antibacterial and anticandida activities

#### 2.4.1 Well diffusion assay

The antibacterial and anticandida activities of *F. aegyptia* and *Z. spinosa* herb extracts were tested as previously recommended against *S. aureus* ATCC 25923, *L. monocytogenes* ATCC 19115, *E. faecalis* ATCC 29212, *E. coli* ATCC 35218, *Salmonella* Typhimurium ATCC 1408, *P. aeruginosa* ATCC 27853, *Candida neoformans* ATCC 14116, *Candida albicans* ATCC 90028, and *Candida krusei* ATCC 6258 (EUCAST 2024; CLSI, 2018). Briefly, the strains were grown in Mueller–Hinton (MH) broth (Oxoid, USA) at 37 °C for 24 h, and suspensions were adjusted to 0.5 McFarland standard turbidity. Then, 100 µL of each precultured suspension was spread onto plates containing MH agar. Wells were cut with a sterile borer (four to eight mm in diameter), and impregnated with 100 µL of the different plant extracts used. Ciprofloxacin and Amphotericin B (Oxoid, USA) served as positive controls. The treated plates were placed at 4 °C for 1 h and then incubated at 37 °C overnight (Memmert, Germany). After incubation, the inhibition zone surrounding the wells was measured. Each sample was tested in triplicate.

To determine the antibacterial and anticandida effects of *F. aegyptia* and *Z. spinosa* herb extracts, the diameter of the transparent zone around the well (zone of inhibition) was measured, and the results were interpreted as follows: not sensitive = diameter ˂8 mm; sensitive = diameter varying between 8 and 14 mm; very sensitive = diameter varying between 15 and 19 mm; extremely sensitive = diameter ≥20 mm.

#### 2.4.2 Minimal inhibitory concentration assay

The minimal inhibitory concentration (MIC) is defined as the lowest concentration showing no visible growth after 24 h incubation. For the MIC assay, the tested extracts of *F. aegyptia* and *Z. spinosa* were diluted to 10,000 μg/mL in a 5% DMSO solution and placed in 96-well microtiter plates (SARSTEDT AG and Co. KG, Numbrecht, Allemagne). It is important to note that the concentration of DMSO used in the tested extracts of the two plants solutions experiments did not exceed 0.05%, which is non-toxic to bacteria (Cirino et al., 2023).

The MIC was determined using the broth microdilution assay as a standard method (EUCAST 2024; CLSI, 2018). Thus, these extracts were then further serially diluted in Mueller-Hinton broth (Biolife, Italy) to achieve concentrations ranging from 10,000 μg/mL down to 312 μg/mL. Next, 10 μL of each bacterial and *candida* selected isolates, adjusted to a 0.5 McFarland turbidity standard, were added to each well, which contained 100 μL of the serially diluted extracts. Finally, 10 µL of resazurin (100 μg/mL) was added to each well as an indicator dye. The microtiter plates were incubated for 24 h at 37 °C (Besbes Hlila et al., 2016).

### 2.5 Minimal bactericidal/fungicidal concentration (MBC/MFC) assay

To estimate the MBC and the Minimum Fungicidal Concentration (MFC) of each herb extracts of *F. aegyptia* and *Z. spinosa*, 10 μL of medium from each well that showed no growth were transferred to Mueller-Hinton agar plates (Biolife, Italy) as previously recommended (EUCAST 2024; CLSI, 2018). These plates were incubated for 24 h at 37 °C, and we examined bacterial and candidal growth after incubation. The concentration at which no microbial growth occurred was identified as the MBC or MFC. To characterize the effectiveness of the analyzed samples, we calculated the MFC/MIC and MBC/MIC ratios. A ratio of less than or equal to four indicated a bactericidal effect, while a ratio greater than four indicated a bacteriostatic action (Hlila et al., 2017).

### 2.6 Antibiofilm assay

#### 2.6.1 Biofilm production test

For the biofilm formation assay among clinical isolates of *S. aureus* and *P. aeruginosa*, we used the microtiter method using 96-well polystyrene microtiter sterile plates as previously recommended (CLSI, 2018). Briefly, clinical isolates were cultured on nutrient agar plates at 37 °C overnight, and 0.5 McFarland standard was prepared from each bacterial sample in phosphate-buffered saline (PBS). Then, 20 µL of bacterial suspension was mixed with 180 µL trypticase soy broth (TSB, Condalab Co., Madrid, Spain) supplemented with 1% glucose and added to sterile 96-well polystyrene microtiter plates. After incubation overnight at 37 °C, the microplates were carefully washed three times with sterile PBS, and then microplates were inverted to dry for 20 min at room temperature. For biofilm quantification, 200 µL of 2% safranin dye solution was added to the well for 40 min at room temperature and washed three times with sterile PBS. Safranin bound to the biofilm in each well was extracted with 200 µL of pure ethanol, and the absorbance of the extracted safranin was measured at 490 nm in an ELISA reader (BioTek, Agilent Technologies, Inc., Santa Clara, CA 95051, USA). Each assay was performed in triplicate. TSB +1% glucose medium was used as a negative control to determine background optical density (OD). The cut-off ODs for biofilm formation were determined as the average OD of the negative control +3 × standard deviation (SD) of the negative control. The OD value was calculated for each microtiter plate separately. OD > 4 × ODc was considered a strong biofilm formation; 2 × ODc < OD ≤ 4 × ODc was considered a moderate biofilm formation; ODc < OD ≤ 2 × ODc was considered a weak biofilm formation; and OD ≤ ODc was considered a nonbiofilm formation.

#### 2.6.2 Biofilm inhibition assay

Briefly, the antibiofilm activity was assessed using the crystal violet test in 96-well polystyrene sterile plates with a flat-bottom microplate format according to according to the conventional guidelines (CLSI, 2018). Five dilutions of the samples (625–10,000 μg/mL) were prepared and combined with a suspension of pathogenic bacteria cultured in brain heart infusion (BHI) for 24 h at 37 °C, at 10^5^ CFU/mL. We used 96 U-bottomed plates containing BHI with 2% glucose (w/v). The positive control consisted of BHI with 2% glucose inoculated with pathogenic bacteria, while the negative control contained only BHI with 2% glucose. After incubation at 37 °C for 24 h, the plates were washed three times with phosphate-buffered saline. The biofilm cells were fixed with methanol for 15 min, air-dried, and stained with 1% crystal violet. Biofilm formation was quantified using a microplate reader, by measuring absorbance (A) at 595 nm. All experiments were performed in triplicate. The percentage of inhibition (%) was calculated using the following formula: % Inhibition = [(A of control − A of extract)/A of control] × 100.

### 2.7 Chemical composition of active extracts

#### 2.7.1 GC-MS analysis

The phytocompounds present in the most active samples were determined by the GC-MS as previously described (Besbes et al., 2025). Briefly, this analysis used a Perkin Elmer Clarus 600 T coupled to a single quadrupole mass spectrometer. An elite 5-M column with helium gas as a carrier, flowing at 1 mL per minute, was employed. The temperature started at 40 °C for 1 minute, then increased to 150 °C at 10 °C per minute, and finally to over 300 °C at the same rate for 3 minutes. The inlet line and ion source temperatures were set to 220 °C and 200 °C, respectively. Ionization occurred at 70 eV, with a mass scanning range of 40–618 m/z. Peaks were identified by comparing them to Wiley sixth. edition mass spectral library, standards, and published data. Percentages of detected compounds were calculated using GC peak areas. Kovats index of each compound was determined using retention times of C6-C26 n-alkanes and compared to literature values (Adams, 2017).

#### 2.7.2 LC-HRMS analysis

The phytochemistry of the extracts was analyzed using a UHPLC-PDA-Detector Mass Spectrophotometer (HR-L = CMS 1290 Infinity UHPLC System, Agilent Technologies®, Santa Clara, CA, USA). The liquid chromatographic system consists of a binary gradient solvent pump, a HiP sampler, a quadrupole time-of-flight mass spectrometer (MS Q-TOF) equipped with a dual Agilent Jet Stream Electrospray (AJS ES) ion source, and a column compartment. A 10 µL sample was injected into the system, followed by separation in an SB-C18 column (2.1 mm × 50 mm, 1.8 µm particle size). The mobile phase consisted of solvent A (1% formic acid in deionized water) and solvent B (acetonitrile). A flow rate of 0.350 mL/min was used for MS detection in the MS Q-TOF. Phytocompounds were identified based on their mass spectra and unique mass fragmentation patterns. The primary tools for identifying the phytochemical products included Compound Discoverer 2.1, ChemSpider, and PubChem (Reddy et al., 2020).

### 2.8 Molecular docking study

In-silico interaction analysis was performed using the AutoDock 4.2 program package (Trott and Olson, 2010). The crystal structure of the target receptor (PDB: 6mki) (Moon et al., 2018) was retrieved from the RCSB Protein Data Bank (https://www.rcsb.org/). The target protein was prepared by removing the co-crystallized ligand and water molecules, and by adding any missing hydrogen atoms and Gasteiger charges. AutoDock Tools were used to save the files for the tested ligands and the target protein (PDBQT format). The optimization of all compound geometries was done using ACD (3D viewer) software (http://www.filefacts.com/acd3d-viewer-freeware-info). At the same time, the visualization and analysis of interactions were conducted using Discovery Studio 2017R2 (https://www.3dsbiovia.com/products/collaborative-science/biovia-discovery-studio/) and PyMOL (The PyMOL Molecular Graphics System, Version 1.2r3pre, Schrödinger, LLC).

### 2.9 Statistical analysis

The antimicrobial and antibiofilm analyses were performed using two tests for each sample. All data were first assessed for normality using a Kolmogorov–Smirnov test. The results were found to be normally distributed (p > 0.05 in the K-S test) and were analyzed using a one-way ANOVA test and expressed as mean values ± standard (mean ± SEM) (SPSS 22). The Duncan test (p < 0.05) was applied to compare the averages.

## 3 Results and discussion

### 3.1 Antibacterial activities of *Farsetia aegyptia* and *Zilla spinosa* herb extracts

Historically, MDR, extensively drug-resistant (XDR), and pan-drug-resistant (PDR) Gram-negative and Gram-positive bacterial strains, such as *E. coli*, *Klebsiella pneumoniae*, *Acinetobacter baumannii*, *P. aeruginosa*, *S. aureus, L. monocytogenes, E. faecalis, Salmonella* Typhimurium, and *Mycobacterium tuberculosis,* have been detected worldwide to have numerous resistance mechanisms (WHO 2024; Srinivas and Rivard, 2017; Tarchouna et al., 2013; Magiorakos et al., 2012; Ben Kahla et al., 2011). Therefore, the world is now facing a significant and growing threat from the rapid emergence of bacteria resistant to almost all available antibiotics (Alibi et al., 2021; Sherry and Howden, 2018; Magiorakos et al., 2012). As highlighted by the Infectious Diseases Society of America in the “Bad Bugs, No Drugs” paper, “as antibiotic discovery stagnates, a public health crisis brews” (Talbot et al., 2006).

According to the literature, due to their multiple bioactive compounds, medicinal plants have been extensively used as an essential source for synthesizing diverse drugs for many decades. In this setting, our studies and others previously reported interesting effects of essential oils extracted from medicinal plants on a broad spectrum of pathogenic and multi-drug-resistant bacterial strains, such as *K. pneumoniae*, *Salmonella enteritidis*, *Corynebacterium striatum*, and *E. coli* (Ben Selma et al., 2025; Alibi et al., 2022; Alibi et al., 2020).

The antibacterial effects of *F. aegyptia* and *Z. spinosa* chloroformic, ethanolic, and water were evaluated by using two methods: the well diffusion method and the microdilution test against three Gram-positive bacterial strains: *S. aureus* ATCC 25923, *L. monocytogenes* ATCC 19115, *E. faecalis* ATCC 29212, and three Gram-negative bacteria: *E. coli* ATCC 35218, *Salmonella Typhimurium* ATCC 14080, and *P. aeruginosa* ATCC 27853.

Table 1 presents the results of the scusseptibility method. This assay revealed that the chloroform extract of *Z. spinosa* exhibited the strongest antibacterial activity, producing inhibition zones of 13.0 ± 0.5 mm against *S. aureus*, 15.0 ± 0.5 mm against *L. monocytogenes*, 15.8 ± 1.33 mm against *E. faecalis*, 12.0 ± 0.5 mm against *E. coli*, and 12.4 ± 0.5 mm against *Salmonella Typhimurium*. The ethanol extract of *F. aegyptia* showed selective activity, with the highest effect against *L. monocytogenes* (14.5 ± 1.33 mm) and moderate inhibition of *E. faecalis* (11.8 ± 0.5 mm). Meanwhile, the ethanol extract of *Z. spinosa* was particularly active against *S. aureus* (16.0 ± 0.5 mm) and moderately effective against *E. faecalis* (13.1 ± 0.33 mm). Both aqueous extracts demonstrated weak activity, with inhibition zones not exceeding 9.5 mm.

**TABLE 1:** Antimicrobial activities of *Farsetia aegyptia* and *Zilla spinosa* extracts by disk method.

Strains	Fa CH	Zs CH	FaET	Zs ET	Fa W	Zs W	Ciprofloxacin	Amphotericin B
	DIZ	DIZ	DIZ	DIZ	DIZ	DIZ	DIZ	
*Staphylococcus aureus* ATCC 25923	12.5 ± 1.33^hi^	13 ± 0.5^h^	6.5 ± 0.5^L^	16 ± 0.5^e^	6 ± 0.8^L^	0.00^m^	25 ± 0.2^a^	-
*Listeria monocytogenes* ATCC 19115	10 ± 0.5^j^	15 ± 0.5^f^	14.5 ± 1.33^g^	12 ± 0.5^hi^	6.7 ± 0.5^L^	0.00^m^	25.3 ± 1.33^a^	-
*Enterococcus faecalis* ATCC 29212	14.2 ± 0.5^g^	15.8 ± 1.33^f^	11.8 ± 0.5^i^	13.1 ± 0.33^h^	9.5 ± 0.5^j^	8.3 ± 1.33^k^	18 ± 0.1^day^	-
*Escherichia coli* ATCC 35218	11.8 ± 0.33^i^	12 ± 0.5^hi^	6.4 ± 0.5^L^	11 ± 0.1^i^	4.8 ± 0.33^lm^	0.00^m^	21.8 ± 0.1^b^	-
*Salmonella* typhimurium ATCC 14080	0.00^m^	12.4 ± 0.5^hi^	7.6 ± 1.33^kL^	0.00^m^	8.1 ± 1.33^k^	7.2 ± 0.33^kL^	19.3 ± 0.33^c^	-
*Pseudomonas aeruginosa* ATCC 27853	7 ± 0.5^kL^	5.3 ± 1.33^lm^	6.8 ± 0.5^L^	8.8 ± 0.33^k^	5 ± 0.5^lm^	0.00^m^	19 ± 0.1^c^	-
*Candida albicans* ATCC 90028	8 ± 0.2^k^	10.5 ± 0.2^j^	9 ± 0.2^k^	10.9 ± 1.33^i^	8.4 ± 0.2^k^	11 ± 1.33^i^	-	13.3 ± 0.2^h^
*Candida krusei* ATCC 6258	13-±0.5^h^	11.4 ± 0.5^i^	8.5 ± 0.2^k^	8.02 ± 1.5^k^	0.00^m^	8 ± 0.5^k^	-	12.8 ± 1.33^hi^
*Candida neoformans* ATCC 14116	7 ± 1.33^kL^	7 ± 0.5^kL^	7.2 ± 1.33^kL^	11.5 ± 0.2^i^	4.3 ± 0.2^lm^	7.2 ± 0.5^kL^	-	13 ± 0.5^h^

DIZ: Diameter Inhibition zone expressed as mm. Fa CH: *farsetia aegyptia* chloroformic sample; Zs CH: *zilla spinosa* chloroformic sample; Fa ET: *farsetia aegyptia* ethanolic sample; Zs ET: *zilla spinosa* ethanolic sample; Fa W: *farsetia aegyptia* water sample; Zs W: *Zilla spinosa* water sample. The letters (a–m) show an important difference between the different doses of extracts in accordance with the Duncan assay (p < 0.05).

Our findings indicate that the water and organic extracts of the two plants, *F. aegyptia* and *Z. spinosa,* exhibit varying degrees of antibacterial activity, with MIC values ranging from 625 μg/mL to over 1,000 μg/mL. Among the extracts tested, the chloroform extracts of these plants showed stronger antibacterial activity. Thus, the chloroform extracts of both *F. aegyptia* and *Z. spinosa* showed the lowest MIC values against all Gram-positive and Gram-negative bacterial strains selected, ranging from 625 μg/mL to 5,000 μg/mL, except for *Salmonella* Typhimurium ATCC 14080, which had a MIC value greater than 1,000 μg/mL (Table 1). Furthermore, it exhibited substantial inhibition against *E. faecalis* ATCC 29212, with both the MBC and MIC at 625 μg/mL. Additionally, they showed significant inhibition against *L. monocytogenes* ATCC 19115 and *S. aureus* ATCC 25923, with MIC values ranging from 625 μg/mL to 2,500 μg/mL. Interestingly, these two extracts exhibit bactericidal effects against *E. faecalis* ATCC 29212 and *L. monocytogenes* ATCC 19115 (MBC/MIC = 1) and bacteriostatic activities against *S. aureus* ATCC 25923 (MBC/MIC >4).

The ethanolic extracts of *Z. spinosa* and *F. aegyptia* also displayed notable antibacterial activity against *S. aureus* ATCC 25923 and *L. monocytogenes* ATCC 19115, with MIC values of 625 μg/mL and 1,250 μg/mL, respectively.

According to the literature, a previous study reported antibacterial activity of aqueous-ethanol and aqueous-methanol extracts derived from the roots and aerial parts of *Z. spinosa* collected from Asir, Saudi Arabia (Suleiman and Ateeg, 2020). Thus, by using the disk diffusion and microdilution assays, they showed that the aqueous methanolic extract of aerial parts of *Z. spinosa* exerted antibacterial effect, especially against *S. aureus* strain, with a zone of inhibition of 26.5 mm and a MIC of 128 μg/mL (Suleiman and Ateeg, 2020). These results were in the same line as our results, which argue for the importance of organic technique extraction of aerial parts of *Z. spinosa*. In another earlier study, Atta and collaborators demonstrated that the antimicrobial activity of methanolic extract of *Farsetia* aegyptia from Sinai, Egypt, was more active against *S. aureus* than against *Salmonella Typhimurium* and *E. coli* by using a sensitivity test, the disk method (Atta et al., 2013). To explain the differences between the results of this study and those of our study, some factors could be implicated, such as the solvents, extraction methods, biotic and abiotic characteristics of the plant species used.

Additionally, other studies have evaluated the antimicrobial properties of different *Farsetia* species (Hayat and Uzair, 2019). Results indicated that the polar methanolic extract was less active than the medium-polar dichloromethane sample, which aligns with our findings, except for *Salmonella Typhimurium*. In this case, the *F. aegyptia* water extract had a lower MIC than the chloroformic sample. An acetone extract of *Farsetia hamiltonii* demonstrated notable effects against *S. aureus* and *P. aeruginosa* (Ejaz et al., 2023). Another *Farsetia* species tested for its antibacterial properties was *Farsetia heliophila*, which was collected from Pakistan. This extract exhibited significant antibacterial activity against the same Gram-negative bacteria examined in our study, confirming that *Salmonella Typhimurium* is the most sensitive among the Gram-negative strains. Our study found that the *F. aegyptia* water extract notably affected *Salmonella Typhimurium*, with a MIC of 2,500 μg/mL (Burki et al., 2022). Furthermore, in the study by Salma et al. (2018), the 80% alcoholic and water extracts of *Farsetia stylosa* and *Farsetia longiliqua* showed no activity against *P. aeruginosa*, *E. coli*, *Salmonella* Typhimurium, and *S. aureus*.

Furthermore, in this study, the aqueous extracts of both plants exhibited a moderate inhibitory effect on Gram-positive and Gram-negative growth, with MIC values ranging from 5,000 μg/mL to more than 10,000 μg/mL. These aqueous samples showed bactericidal effects against *E. faecalis* ATCC 29212 and *L. monocytogenes* ATCC 19115 (MBC/MIC = 1).

Moreover, the disk diffusion assay showed that the aqueous extract of *Z. spinosa* exhibited no detectable antibacterial activity, whereas the ethanolic extract produced a moderate inhibition zone of 16 mm. This variation may be explained by the differences in the solubility of bioactive constituents, suggesting that the synergistic interaction of ethanol and water extracted compounds could yield a more pronounced antibacterial effect than their individual activities.

It is important to indicate that in this study, practically all tested chloroformic, ethanolic and aqueous extracts of the plants *Z. spinosa* and Farsetia *aegyptia* used significantly affected Gram-positive bacteria more than Gram-negative strains. This difference of action of these extracts could be explained by one or more of these factors: i) the difference in phytochemical composition of different extracts used of these plants, and ii) the high sensitivity of Gram-positive bacteria as explained and reported by previous studies (Cosentino et al., 1999; Soković et al., 2007). Thus, this sensitivity is primarily due to the composition of their membranes. In contrast, Gram-negative strains possess double membranes that act as a protective barrier against antibacterial agents.

### 3.2 Anticandida effects of *Farsetia aegyptia* and *Zilla spinosa* herb extracts

The *F. aegyptia* and *Z. spinosa* chlorophormic, ethanolic, and water extracts were evaluated against three strains of *Candida*: *Candida albicans* ATCC 90028, *Candida krusei* ATCC 6258, and *Candida neoformans* ATCC 14116. Table 1 presents the results of the disk diffusion method. The chloroform extract of *F. aegyptia* showed the highest activity, recording 13.0 ± 0.5 mm against *Candida krusei*, comparable to that of amphotericin B (12.8 ± 1.33 mm).

The ethanol extract of *Z. spinosa* also demonstrated consistent antifungal potential, with inhibition zones of 10.9 ± 1.33 mm against *Candida albicans* and 11.5 ± 0.2 mm against *Candida neoformans*. In comparison, the water extract of *Z. spinosa* showed moderate activity against *Candida albicans* (11.0 ± 1.33 mm). Overall, *Z. spinosa* chloroform extract was the most potent antibacterial agent, *F. aegyptia* chloroform extract was the strongest antifungal, and *Z. spinosa* ethanol extract exhibited the broadest antimicrobial spectrum. Thus, we suggest that the heterogeneity of anticandida effects of these plants’ different extracts could be related to differences between their phytochemical compositions.

Table 2 presents the MIC and MFC values obtained for chloroformic, ethanolic, and water extracts of *F. aegyptia* and *Z. spinosa* against the three *Candida* strains. Our results demonstrated that MIC values ranged from 625 μg/mL to over 10,000 μg/mL, while MFC values ranged from 1,250 μg/mL to over 10,000 μg/mL. The chloroformic extract of *F. aegyptia* demonstrated the most significant anticandidal activity against *Candida albicans* ATCC 90028 and *Candida krusei* ATCC 6258, exhibiting the lowest MIC values of 625 μg/mL. This value was followed by the *Z. spinosa* extract, which had MIC values of 1,250 μg/mL for these two *Candida* species.

**TABLE 2:** Antimicrobial effects of *Farsetia aegyptia* and *Zilla spinosa* extracts determined by microdilution method.

Bacteria	Fa CH			Zs CH			Fa ET			Zs ET			Fa W			Zs W			Ciprofloxacin		
	MIC*	MBC**	MBC/MIC ratio	MIC	MBC	MBC/MIC ratio	MIC	MBC	MBC/MIC ratio	MIC	MBC	MBC/MIC ratio	MIC	MBC	MBC/MIC ratio	MIC	MBC	MBC/MIC ratio	MIC	MBC	MBC/MIC ratio
*S. a*	625 ± 1.25^e^	10,000 ± 4.23^i^	16	1,250 ± 2.1^f^	>10,000	-	10,000 ± 5.2^i^	>10,000	-	625 ± 0.2^e^	10,000 ± 2.6^i^	16	10,000 ± 3.2^i^	>10,000	-	>10,000	>10,000	-	31 ± 0.2^a^	125 ± 0.1^c^	4
*L. m*	2,500 ± 4.2^g^	2,500 ± 2.1^g^	1	1,250 ± 2.08^f^	1,250 ± 1.3^f^	1	1,250 ± 0.36^f^	1,250 ± 0.36^f^	1	2,500 ± 2.3^g^	2,500 ± 1.3^g^	1	10,000 ± 3.4^i^	10,000 ± 2.3^i^	1	>10,000	>10,000	-	31 ± 0.3^a^	125 ± 0.2^c^	4
*E. f*	625 ± 1.2^e^	625 ± 0.39^e^	1	625 ± 0.68^e^	625 ± 1.7^e^	1	2,500 ± 2.9^g^	2,500 ± 0.98^g^	1	2,500 ± 1.2^g^	500 ± 2.5^g^	1	5,000 ± 2.1^h^	5,000 ± 1.3^h^	1	5,000 ± 2^h^	5,000 ± 3^h^	1	125 ± 0.1^c^	>125	-
*E. c*	5,000 ± 3.5^h^	>10,000	-	5,000 ± 2.69^h^	>10,000	-	10,000 ± 5.9^i^	10,000 ± 3.65^i^	1	5,000 ± 2.6^h^	>10,000	-	>10,000	>10,000	-	>10,000	>10,000	-	62 ± 0.2^b^	62 ± 0.3^b^	1
*S*. T	>10,000	>10,000	-	5,000 ± 3.06^h^	>10,000	-	10,000 ± 4.9^i^	>10,000	-	>10,000	>10,000	-	2,500 ± 0.8^g^	>10,000	-	5,000±2^h^	>10,000	-	125 ± 0.1^c^	125 ± 0.2^c^	1
*P*. *a*	5,000 ± 3.4^h^	>10,000	-	10,000 ± 2.35^i^	>10,000	-	10,000 ± 4.98^i^	>10,000	-	5,000 ± 2.9^h^	>10,000	-	10,000 ± 3.1^i^	>10,000	-	>10,000	>10,000	-	125 ± 0.3^c^	>125	-
*Candida*	MIC	MFC***	MBC/MFC ratio	MIC	MFC	MFC/MIC ratio	MIC	MFC	MFC/MIC ratio	MIC	MFC	MFC/MIC ratio	MIC	MFC	MFC/MIC ratio	MIC	MFC	MFC/MIC ratio	Amphotericin B		
																			MIC		
*C*. *a*	625 ± 2.61^e^	5,000 ± 2.63^h^	8	1,250 ± 0.69^f^	5,000 ± 3.5^h^	4	1,250 ± 0.97^i^	>10,000	-	1,250 ± 0.28^i^	>10,000	-	5,000 ± 2.3^h^	5,000 ± 2.4^h^	1	1,250 ± 0.36^i^	1,250 ± 0.5^i^	1	500 ± 0.5^days^		
*C*. *k*	625 ± 0.9^e^	>10,000	-	1,250 ± 0.98^f^	10,000 ± 2.5^i^	8	2,500 ± 1.65^f^	>10,000	-	5,000 ± 2.9^h^	>10,000	-	>10,000	>10,000	-	5,000 ± 2.1^h^	>10,000	-	500 ± 0.6^days^		
*C. n*	2,500 ± 2.61^g^	>10,000	-	5,000 ± 2.36^h^	>10,000	-	2,500 ± 1.69^f^	>10,000	-	1,250 ± 0.2^i^	10,000 ± 3.4^i^	8	10,000 ± 4.3^i^	>10,000	-	5,000 ± 2.3^h^	10,000 ± 2.5^i^	2	500 ± 0.41^day^		

MIC* : minimal inhibitory concentration is mentioned in μg/mL; MBC** : minimal bactericidal concentration is mentioned in μg/mL, MFC*** : minimal fungicidal concentration is mentioned in μg/mL. Fa CH: *farsetia aegyptia* chloroformic sample; Zs CH: *zilla spinosa* chloroformic sample; Fa ET: *farsetia aegyptia* ethanolic sample; Zs ET: *zilla spinosa* ethanolic sample; Fa W: *farsetia aegyptia* water sample; Zs W: *Zilla spinosa* water sample. *S.a*: *Staphylococcus aureus* ATCC, 25923, *L.m: Listeria monocytogenes* ATCC, 19115, *E.f*: *Enterococcus faecalis* ATCC, 29212, *E.c: Escherichia coli* ATCC, 35218, *S.T: Salmonella Typhimurium* ATCC, 1408, *P.a: Pseudomonas aeruginosa* ATCC, 27853, *C.a: Candida albicans* ATCC, 90028, *C.k: Candida krusei* ATCC, 6258, *C.n: Candida neoformans* ATCC, 14116. The letters (a–i) show an important difference between the different doses of extracts in accordance with the Duncan assay (p < 0.05).

The *F. aegyptia* chloroformic extract showed a fungistatic effect against *Candida albicans* ATCC 90028, with an MFC/MIC ratio of 8. In contrast, the *F. aegyptia* aqueous sample, *Z. spinosa* chloroformic sample, and *Z. spinosa* aqueous extract exhibited a fungicidal effect against this yeast, with MFC/MIC ratios of four or lower. Notably, the *Z. spinosa* extract demonstrated fungicidal activity against *Candida neoformans* ATCC 14116, with an MFC/MIC ratio of 2, whereas the *Z. spinosa* ethanolic sample displayed a fungistatic effect against this strain, with an MFC/MIC ratio of 8.

In a previous study, organic extracts of *Z. spinosa* obtained using solvents such as methyl alcohol, acetone, ethyl acetate, methylene chloride, and petroleum ether were evaluated for their antifungal activity against *Candida albicans* (Alghanem et al., 2018). The authors reported that this *Candida* demonstrated moderate sensitivity to the ethyl acetate, acetone, and methyl alcohol extracts. This finding aligns with our results, as the chloroform, ethanolic, and water samples of this plant had an important effect on *Candida albicans*. However, these results were in disagreement with those of two other studies, which reported no antifungal effects of the methanol extract of *Z. spinosa* collected from Riyadh, Saudi Arabia (Shahat et al., 2017), and the chloroform extract of *Z. spinosa* collected from the Suez-Cairo desert road in Egypt (El-Sharkawy et al., 2013). The heterogeneity of results reported in our study and other studies could be related to one or more of the following factors: solvents used, extraction methods, and environmental effects on the plant specimens collected.

To explain the antibacterial, antifungal, and antibiofilm activities of the organic extracts of the two plants used in the current study, several previous studies have evaluated the precise mechanisms of action of the major compounds in these extracts. In this setting, El-Toumy and his collaborators have demonstrated that both stigmasterol and β-sitosterol, which are the primary compounds in the chloroform extract of the aerial parts of *Z. spinosa* collected in Egypt exhibit antimicrobial properties against *S. aureus* (El-Toumy et al., 2011). Moreover, other studies have shown that stigmasterol, in particular, has important antimicrobial effects against *Candida albicans*, *P. aeruginosa*, *S. aureus*, and *E. coli* (Odiba et al., 2014; Yinusa et al., 2014; Mailafiya et al., 2018).

Moshi and coworkers indicated that a dichloromethane extract of *Uvaria scheffleri* contains a mixture of stigmasterol and β-sitosterol, which is active against *Candida albicans* (Moshi et al., 2004). The antimicrobial activity of these steroids is attributed to their ability to inhibit the enzyme known as 'sortase,' which plays a role in the secretion and anchoring of cell wall proteins (Kanokmedhakul et al., 2005). Additionally, it was reported that disruption of the cell membrane may also be a potential mechanism for the action of steroids on bacteria and fungi (Tamokou et al., 2011).

Palmitic acid was identified as the dominant compound in the chloroform extract of *Z. spinosa*, and exhibited bactericidal activity against *E. faecalis*, with a minimal inhibitory concentration (MIC) of 2 μg/mL (Cheung Lam et al., 2016). Several studies have highlighted the effective antibacterial properties of linolenic acid, a prominent compound in the chloroform extract of *F. aegyptia* (12.68%), particularly against *S. aureus* (Kusumah et al., 2020; Butt et al., 2016). Additionally, it was reported that long-chain fatty acids, such as arachidonic acid, linoleic acid, and oleic acid found in both *F. aegyptia* and *Z. spinosa* extracts, showed interesting antibacterial activities against *S. aureus* (Zheng et al., 2005). Additionally, 13-docosenamide, a fatty amide, and phytol, an acyclic monounsaturated diterpene alcohol also possess antimicrobial properties (Islam et al., 2018; Kumaradevan et al., 2015; Ghaneian et al., 2015).

### 3.3 Anti-biofilm activities of *Farsetia aegyptia* and *Zilla spinosa*

Microbial biofilms consist of colonies of microorganisms embedded in a polysaccharide matrix, adhering to surfaces. They pose substantial biological threats across various settings, including food, drinking water, industrial environments, and clinical settings, as the physical barriers formed by these biofilms hinder the access of antimicrobials to their target sites (Yasbolaghi Sharahi et al., 2025).

Recently, there has been increasing interest in exploring the processes involved in inhibiting bacterial biofilm growth and formation. The initial step in most biofilm formation is attachment. Consequently, the anti-adhesive properties of natural compounds have become a focal point of research aimed at preventing microcolony formation (Kungwani et al., 2024; Alibi et al., 2021). In this setting, the current study investigated the inhibitory effects of chloroform, ethanol, and water extracts from *F. aegyptia* and *Z. spinosa*, both members of the Brassicaceae family, on the adhesion of three Gram-positive bacteria, three Gram-negative bacteria, and three *Candida* strains. Thus, Extracts that demonstrated more than 50% inhibition of biofilm formation are classified as having a good anti-biofilm (ABF) effect. In contrast, those that exhibited less than 50% inhibition are considered to have a weak ABF effect (Sandasi et al., 2008).

Importantly, in the current study, all Gram-positive bacterial strains used, including *S. aureus* ATCC 25923, *L. monocytogenes* ATCC 19115, and *E. faecalis* ATCC 29212; Gram-negative bacterial strains comprising *E. coli* ATCC 35218, *Salmonella* Typhimurium ATCC 1408, and *P. aeruginosa* ATCC 27853, and strains of *Candida*: *Candida neoformans* ATCC 14116, *Candida albicans* ATCC 90028, and *Candida krusei* ATCC 6258, were considered biofilm producers according to the microtiter method results.

The term MBIC50 refers to the minimum biofilm inhibition concentration required to achieve 50% inhibition. This term indicates the lowest antimicrobial dose that produce a ≥50% reduction in biofilm formation (Genovese et al., 2021).

**FIGURE 2:**
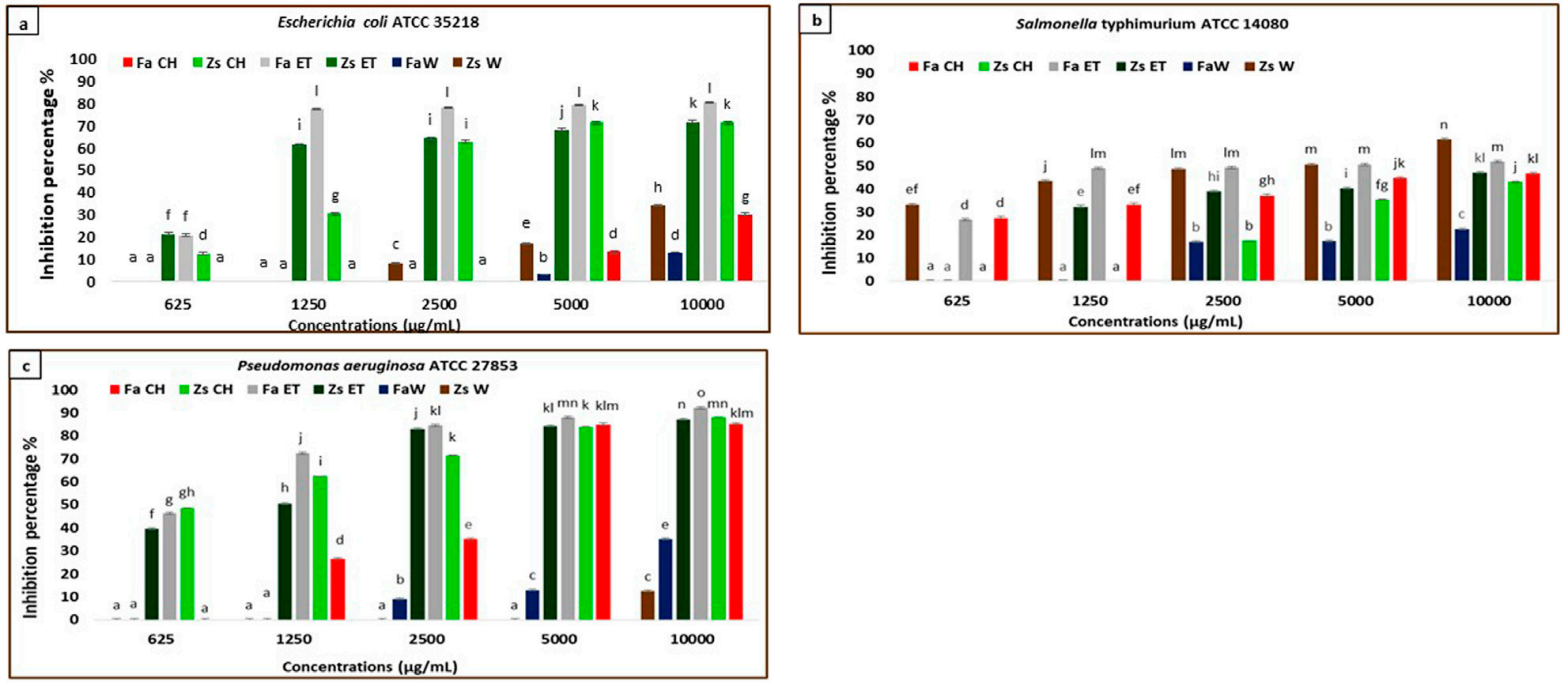
Anti-biofilm activity of *F. aegyptia* chloroformic sample (Fa CH), *Z. spinosa* chloroformic sample (Zs CH), *F. aegyptia* ethanolic sample (Fa ET), *Z. spinosa* ethanolic sample (Zs ET), *F. aegyptia* water sample (Fa W), and *Z. spinosa* water sample (Zs W), against **(a)** Escherichia coli ATCC 35218, **(b)** *Salmonella Typhimurium* ATCC 14080, and **(c)** *Pseudomonas aeruginosa* ATCC 27853. The letters (a -p) on the bars present important differences (p < 0.05).

**FIGURE 3:**
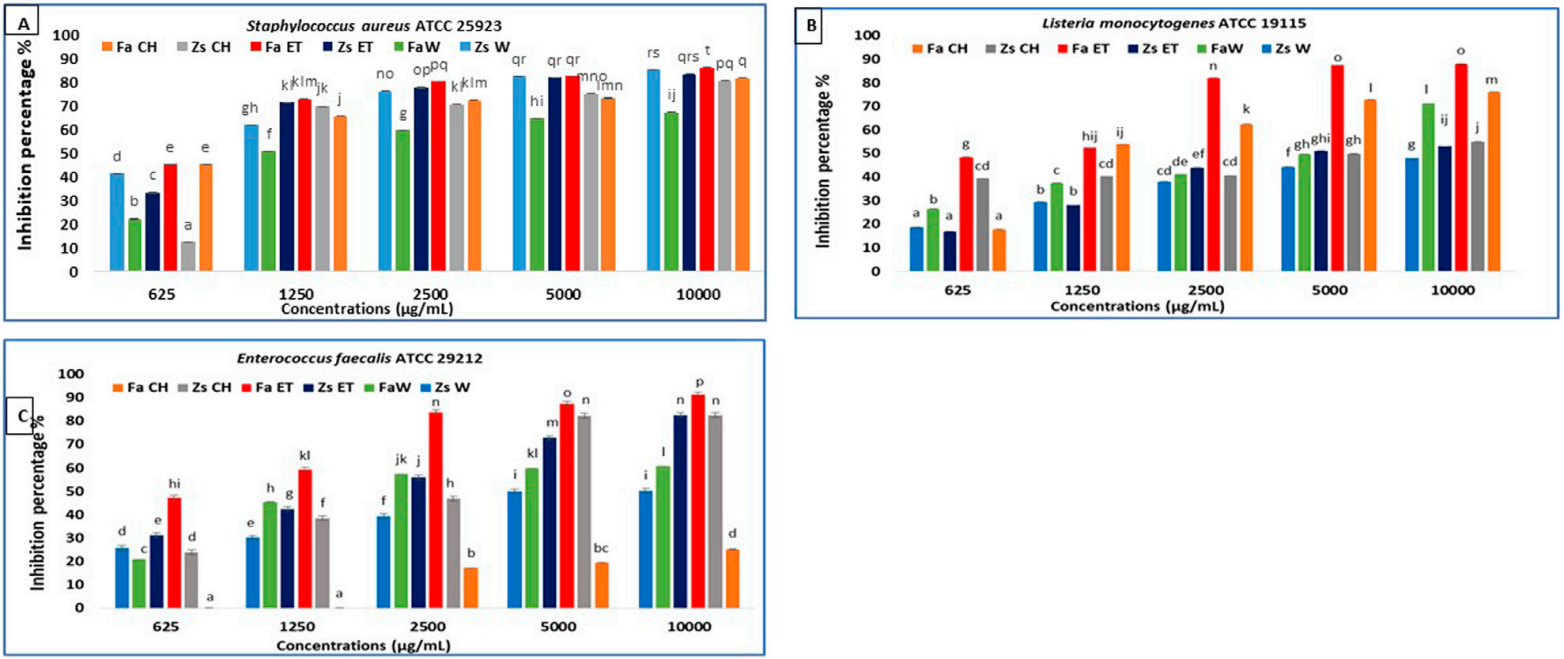
Anti-biofilm activity of *F. aegyptia* chloroformic sample (Fa CH), *Z. spinosa* chloroformic sample (Zs CH), *F. aegyptia* ethanolic sample (Fa ET), *Z. spinosa* ethanolic sample (Zs ET), *F. aegyptia* water sample (Fa W), and *Z. spinosa* water sample (Zs W), against **(A)** Staphylococcus aureus ATCC 25923, **(B)** *Listeria monocytogenes* ATCC 19115, and **(C)** Enterococcus faecalis ATCC 29212. The letters (a -p) on the bars present important differences (p < 0.05).

**FIGURE 4:**
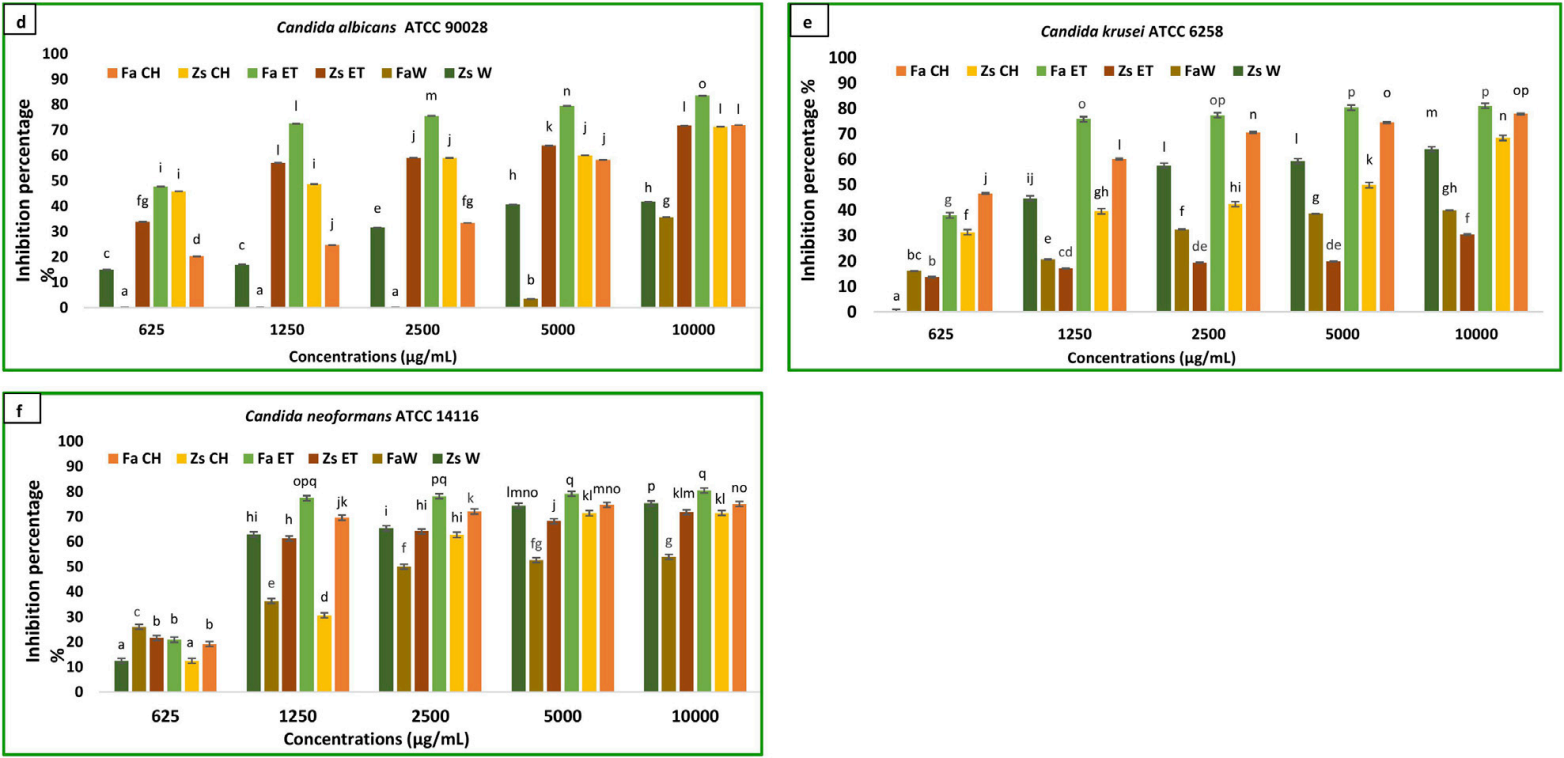
Anti-biofilm activity of F. aegyptia chloroformic sample (Fa CH), Z. spinosa chloroformic sample (Zs CH), F. aegyptia ethanolic sample (Fa ET), Z. spinosa ethanolic sample (Zs ET), F. aegyptia water sample (Fa W), and Z. spinosa water sample (Zs W) against **(d)** *Candida* albicans ATCC 90028, **(e)** *Candida* Krusei ATCC 6258, and **(f)** *Candida* neoformans ATCC 14116. The letters (a–p) on the bars present important differences (p < 0.05).

In the current study, the MBIC50 values are presented in Table 3. The results indicate that the samples effectively inhibit biofilm formation by the selected pathogens when compared to the negative control used in the experiment (Figures 2–4). Notably, the antibiofilm activity of the two plants samples against the tested microorganisms is dose-dependent, with the percentage inhibition increasing as sample concentration increases (p < 0.05). To our knowledge, the current study is the first to show these findings.

**TABLE 3:** Minimal biofilm inhibitory concentration (MBIC) of *Farsetia aegyptia* and *Zilla spinosa* chloroformic, ethanolic, and water samples against Gram-positive, Gram-negative, and *Candida* strains.

Extracts	Fa CH	Zs CH	Fa ET	Zs ET	Fa W	Zs W
Gram-positive bacteria	MBIC50 (μg/mL)					
*Staphylococcus aureus* ATCC 25923	700 ± 0.01^abc^	940 ± 0.89^abcde^	710 ± 0.79^abc^	840 ± 0.21^abc^	1,200 ± 0.31^def^	840 ± 0.05^abc^
*Enterococcus faecalis* ATCC 29212	>10,000	2,660 ± 0.21^jk^	750 ± 0.78^abc^	1820 ± 0.23^i^	1,530 ± 0.22^ghi^	5,050 ± 0.04^n^
*Listeria monocytogenes* ATCC 19115	1,650 ± 0.16^hi^	5,010 ± 0.32^n^	1,020 ± 0.32^bcde^	3,840 ± 0.54^m^	5,020 ± 0.25^n^	>10,000
Gram-negative bacteria	MBIC50 (μg/mL)					
*Pseudomonas aeruginosa* ATCC 27853	3,100 ± 0.03^L^	630 ± 0.25^a^	700 ± 0.24^ab^	1,250 ± 0.33^efg^	>10,000	>10,000
*Escherichia coli* ATCC 35218	>10,000	1820 ± 0.33^i^	870 ± 0.35^abcd^	970 ± 0.34^abcde^	>10,000	>10,000
*Salmonella typhimurium* ATCC 14080	>10,000	>10,000	2,800 ± 0.24^jkl^	>10,000	>10,000	2,850 ± 0.95^kL^
Candida strains	MBIC50 (μg/mL)					
*Candida albicans* ATCC 90028	3,900 ± 0.5^m^	1,350 ± 0.05^fgh^	660 ± 0.65^a^	970 ± 0.24^abcde^	>10,000	>10,000
*Candida krusei* ATCC 6258	750 ± 1.23^abc^	5,100 ± 0.3^n^	770 ± 0.42^abc^	>10,000	>10,000	1,650 ± 0.32^hi^
*Candida neoformans* ATCC 14116	920 ± 1.33^abcde^	1850 ± 0.09^i^	970 ± 0.3^abcde^	960 ± 0.32^abcde^	2,500 ± 0.54^j^	1,000 ± 0.24^bcde^

Fa CH: *farsetia aegyptia* chloroformic sample; Zs CH: *zilla spinosa* chloroformic sample; Fa ET: *farsetia aegyptia* ethanolic sample; Zs ET: *zilla spinosa* ethanolic sample; Fa W: *farsetia aegyptia* water sample; Zs W: *Zilla spinosa* water sample. The letters (a–n) demonstrate a significant difference between the different samples by the Duncan test (p < 0.05).

#### 3.3.1 Gram-negative bacteria anti-biofilm assay

The effects of different extracts of the two plants used in this study on inhibiting biofilm formation against the Gram-negative strains: *E. coli* ATCC 35218, *Salmonella Typhimurium* ATCC 14080, and *P. aeruginosa* ATCC 27853 were indicated in Figures 2a–c. The chloroformic and ethanolic extracts of *Z. spinosa,* and *Z. spinosa,* and the chloroformic extract of *Z. spinosa* showed significant biofilm inhibition against *E. coli* ATCC 35218 (p < 0.05). The most effective sample was the ethanolic extract of *F. aegyptia*, which exhibited biofilm inhibition percentages of 77.38% at 1,250 μg/mL, 78.14% at 2,500 μg/mL, 79.08% at 5,000 μg/mL, and 80.44% at 10,000 μg/mL (Figure 2a) with an MBIC50 of 870 ± 350 μg/mL.

Regarding the second g-negative bacterium, *Salmonella Typhimurium* ATCC 14080, the tested samples showed moderate biofilm inhibition, ranging from 22.65% (for the water extract of *F. aegyptia*) to 61.44% (for the aqueous sample of *Z. spinosa*) at a concentration of 10,000 μg/mL (Figure 2b) (p < 0.05). At both 10,000 μg/mL and 5,000 μg/mL, *Z. spinosa* and *F. aegyptia*, including their chloroformic and ethanolic extracts, demonstrated considerable biofilm inhibition against *P. aeruginosa* ATCC 27853 (greater than 83%) (Figure 2c), with MBIC50 values ranging from 630 ± 250 μg/mL to 3,100 ± 30 μg/mL (Table 3) (p < 0.05).

#### 3.3.2 Gram-positive bacteria anti-biofilm assay

The treatment of *S. aureus* ATCC 25923 with *F. aegyptia* and *Z. spinosa* samples for 24 h resulted in a decrease in biofilm formation across all concentrations tested (1,000 μg/mL, 5,000 μg/mL, 2,500 μg/mL, 1,250 μg/mL, and 625 μg/mL). For the highest concentration of 10,000 μg/mL, all tested samples of these plants extracts showed significant inhibition of biofilm formation, with more than 80% inhibition. However, the *F. aegyptia* water extract showed a lower inhibition rate of 67.49% (Figure 3A). The best MBIC50 values were observed at 700 ± 10 μg/mL for the *F. aegyptia* chloroform extract and at 710 ± 790 μg/mL for the *F. aegyptia* ethanolic extract (Table 3).

For the second g-positive bacterium, *L. monocytogenes* ATCC 19115, extracts from *F. aegyptia* were more effective than those from *Z. spinosa*. Notably, the ethanolic extract exhibited the best antibiofilm effect, achieving 87.94% inhibition at 10,000 μg/mL, 87.53% at 5,000 μg/mL, and 82.02% at 2,500 μg/mL (Figure 3B). The MBIC50 for this extract was 1,020 ± 320 μg/mL, followed by the chloroform extract, which showed a 76.02% inhibition at 10,000 μg/mL with an MBIC50 of 1,650 ± 160 μg/mL (Table 3). For *E. faecalis* ATCC 29212, the *F. aegyptia* ethanolic extract demonstrated more than 91% inhibition at 10,000 μg/mL. The chloroform and ethanol extracts of *Z. spinosa* also significantly inhibited biofilm formation, with inhibition rates of 82.4% and 82.61%, respectively (Figure 3C). In contrast, the *F. aegyptia* chloroform extract exhibited weak inhibition against *E. faecalis*, with only 25.06% inhibition at 10,000 μg/mL.

The formation of biofilm plays an important role in pathogenesis, and biofilm development is mediated by a signal-based quorum-sensing system (Yasbolaghi Sharahi et al., 2025; Kungwani et al., 2024; Alibi et al., 2020). Hens, biofilm infections have become a threat in injured and post-surgical patients, leading to prolonged wound healing as well as high patient morbidity and mortality.

In the early stages of the wound, the host immune system destroys microbes. However, when these microbes adhere to the wound surface and cells, they multiply rapidly through quorum sensing, thereby forming biofilms (Yasbolaghi Sharahi et al., 2025; Kungwani et al., 2024). The well-formed biofilm develops resistance (1,000 times more) to destruction by the host immune system and antibiotics (Kungwani et al., 2024). In this setting, the current study showed reduced biofilm biomass production in Gram-positive, Gram-negative, and *Candida* strains when treated with chloroformic or ethanolic extracts of *Z. spinosa* and *F. aegyptia*. MBIC50 was strain-dependent, showing different resistance levels against these extracts. Moreover, we suggest that compounds from these antimicrobial extracts could have disrupted the mechanism of attachment and the formation of the extracellular polymeric substance (EPS), or interfered with the maturation stage of biofilm formation and the quorum sensing system, thereby preventing the development of bacterial biofilms.

It is well established that biofilm resistance to antibiotics is due to intrinsic structural properties, such as limited antibiotic permeability through the EPS, the production of efflux pumps, and the release of inactivating enzymes. They may acquire resistance through gene expression and mutational changes, obstructing antibiotic permeation, inducing slow growth and adaptive stress responses, and leading to persistent cell proliferation (Gedefie et al., 2023; Garousi et al., 2022; Zhao et al., 2020; Hosseini et al., 2020). Biofilms exhibit varying physiological and functional properties; thus, a combination of therapeutics could be better than a single antibiotic intervention for managing and treating biofilm infections (Yasbolaghi Sharahi et al., 2025; Kungwani et al., 2024).

Hence, recent research has focussed on exploring plants as a potential therapeutic solution to biofilm infections. Plants contain bioactive phytochemicals that work together to prevent antibiotic resistance with minimal side effects (Yasbolaghi Sharahi et al., 2025; Kungwani et al., 2024; Silva et al., 2023).

In this context, even the chloroformic or ethanolic extracts of the two plants used in this study showed significant antibiofilm activity against Gram-positive, Gram-negative, and *Candida* strains, but the MBIC50 values were considerably high. These MBIC50 values were approximately similar to those of previous MBIC50 levels for standard antibiotics (Ferreira et al., 2024; Silva et al., 2023). Thus, to reduce the MBIC50 values of the organic extracts in this study, additional studies will be needed to evaluate the combined use of these extracts or their major compounds with conventional antibiotics to inhibit biofilm formation.

#### 3.3.3 *Candida* antibiofilm assay

The findings regarding the *in vitro* antibiofilm effects of different *Z. spinosa* and *F. aegyptia* extracts against three *Candida* species are illustrated in Figures 4d–f. For a concentration of 10,000 μg/mL, the ethanolic extract of *F. aegyptia* inhibits 83.52% of biofilm formation of *Candida albicans* ATCC 90028. Additionally, the MBIC50 was determined to be 660 ± 650 μg/mL, which resulted in a 50% reduction in biofilm formation (Table 3). The chloroform extracts from the two tested plants, along with the ethanolic extract of *Z. spinosa*, also demonstrated significant antibiofilm activity, exceeding 71% against this yeast (p < 0.05). Conversely, the water extracts of *Z. spinosa* and *F. aegyptia* exhibited weak biofilm inhibition rates of 35.64% and 41.74%, respectively (Figure 4d). The impact on biofilm formation varies among the different *Candida* strains tested. The ethanolic extract of *Z. spinosa* showed similar effects on biofilm formation of both *Candida albicans* ATCC 90028 and *Candida neoformans* ATCC 14116, with inhibition percentages of 71.73% and 71.77%, respectively (Figures 4d,f). However, this extract had a limited effect on biofilm formation by *Candida Krusei* ATCC 6258, with an inhibition percentage of only 30.41% (Figure 4e). In contrast, the ethanolic extract of *F. aegyptia* significantly inhibited biofilm formation by all three *Candida* strains, achieving over 80% inhibition. Overall, our results indicate that organic extracts significantly inhibit biofilm formation of *Candida* strains compared to aqueous extracts, except for the *Z. spinosa* water extract against *Candida neoformans* ATCC 14116, which demonstrated an inhibition rate of 75.25%. The MBIC50 values ranged from 660 ± 650 μg/mL to more than 10,000 μg/mL (Table 3).

### 3.4 Chemical composition of *Farsetia aegyptia* and *Zilla spinosa* chloroformic extract by GC-MS and LC-HRMS

**FIGURE 5:**
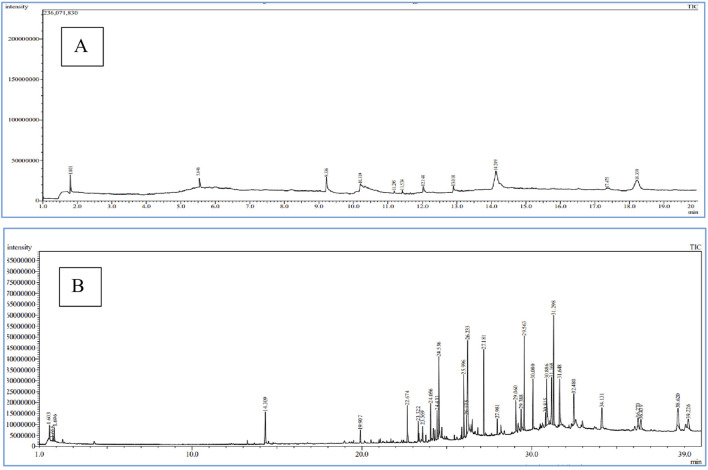
Chromatograms of Z. spinosa **(A)** and F. aegyptia **(B)** chloroform extracts by GC-MS method.

In this study, we selected chloroform extracts from *F. aegyptia* and *Z. spinosa* for qualitative analysis using GC-MS due to their notable antimicrobial properties. The goal of this experiment is to identify the pharmacologically active constituents present in these extracts. This technique involves comparing retention times, molecular formulas, and other relevant factors. Our findings indicate that both non-polar and polar compounds are present, with a total of 28 phytocomponents identified. Figures 5a,b show the chromatograms of the two chloroform extracts, and the detected compounds are summarized in Table 4. Each compound is described with its molecular formula, retention time, structural information, and area percentages. The chloroform extract of *Z. spinosa* includes 10 compounds, such as: Phytosterols: β-sitosterol (40.39%) and stigmasterol (22.24%) - Benzopyrone: coumarin (9.25%) - Fatty acids: n-decanoic acid (6.40%), arachidonic acid (3.73%), oleic acid (3.73%), and hexanoic acid (3.38%) - Terpene: neophytadiene (1.24%) - Benzofuran: loliolide (0.88%) - Terpenoid: cyclolaudenol (3.73%). This comprehensive analysis highlights the rich phytochemical profile of these extracts.

**TABLE 4:** Components detected in the chloroform samples of *Farsetia aegyptia* and *Zilla spinosa* by GC-MS.

Compound number	Compounds	Class	Molecular formula	Retention time	Area percentage %	
					FaCH	ZsCH
1	Pentanoic acid, 3-methyl-4-oxo-	Fatty acid esters	C6H10O3	1.696	3.85	
2	Hexanoic acid	Fatty acid	C6H12O2	5.646		3.38
3	Coumarin	Chromenones	C9H6O2	9.336		9.25
4	n-Decanoic acid	Fatty acid	C10H20O2	10.319		6.40
5	Loliolide	Benzofurans	C11H16O3	11.295	3.70	0.88
6	Neophytadiene	Terpene	C20H38	11.534	1.65	1.24
7	Arachidonic acid	Fatty acid	C20H40O2	12.144	6.96	3.73
8	Oleic acid	Fatty acid	C18H34O2	13.014		3.73
9	Beta-sitosterol	Phytosterol	C29H50O	14.249		40.39
10	Naphtalene	Naphthalenes	C10H8	14.309	3.76	
11	Cyclolaudenol	Steroid	C31H52O	17.475		3.73
12	Stigmasterol	Phytosterol	C29H48O	18.339	1.84	22.24
13	2 (4H)-benzofuranone, 5,6,7,7a-tetrahydro-4,4,7a-trimethyl-, (R)-	Benzofurans	C11H16O2	19.907	0.82	
14	1,2-Benzenedicarboxylic acid, bis (2-methylpropyl) ester	Benzoic acid esters	C16H22O4	24.056	3.41	
15	Phytol	Alcohol	C20H40O	25.996	5.6	
16	Linoleic acid	Fatty acid	C18H32O2	26.175	1.6	
17	Linolenic acid	Fatty acid	C18H30O2	26.233	12.68	
18	Hexadecanoic acid, 2-hydroxy-1-(hydroxymethyl) ethyl ester	Fattyacidethylesterofglycerol	C19H38O4	29.388	1.88	
19	Linoleic acid ethyl ester	Fatty acid esters	C20H36O2	30.815	1.15	
20	Linolenic acid, ethyl ester	Fatty acid esters	C20H34O2	30.886	7.31	
21	13-Docosenamide, (Z)-	Fatty amides	C22H43NO	31.648	6.47	
22	Cholesterol	Steroid	C27H46O	36.439	4.64	
23	Campesterol	Phytosterol	C28H48O	38.620	2.17	

**FIGURE 6:**
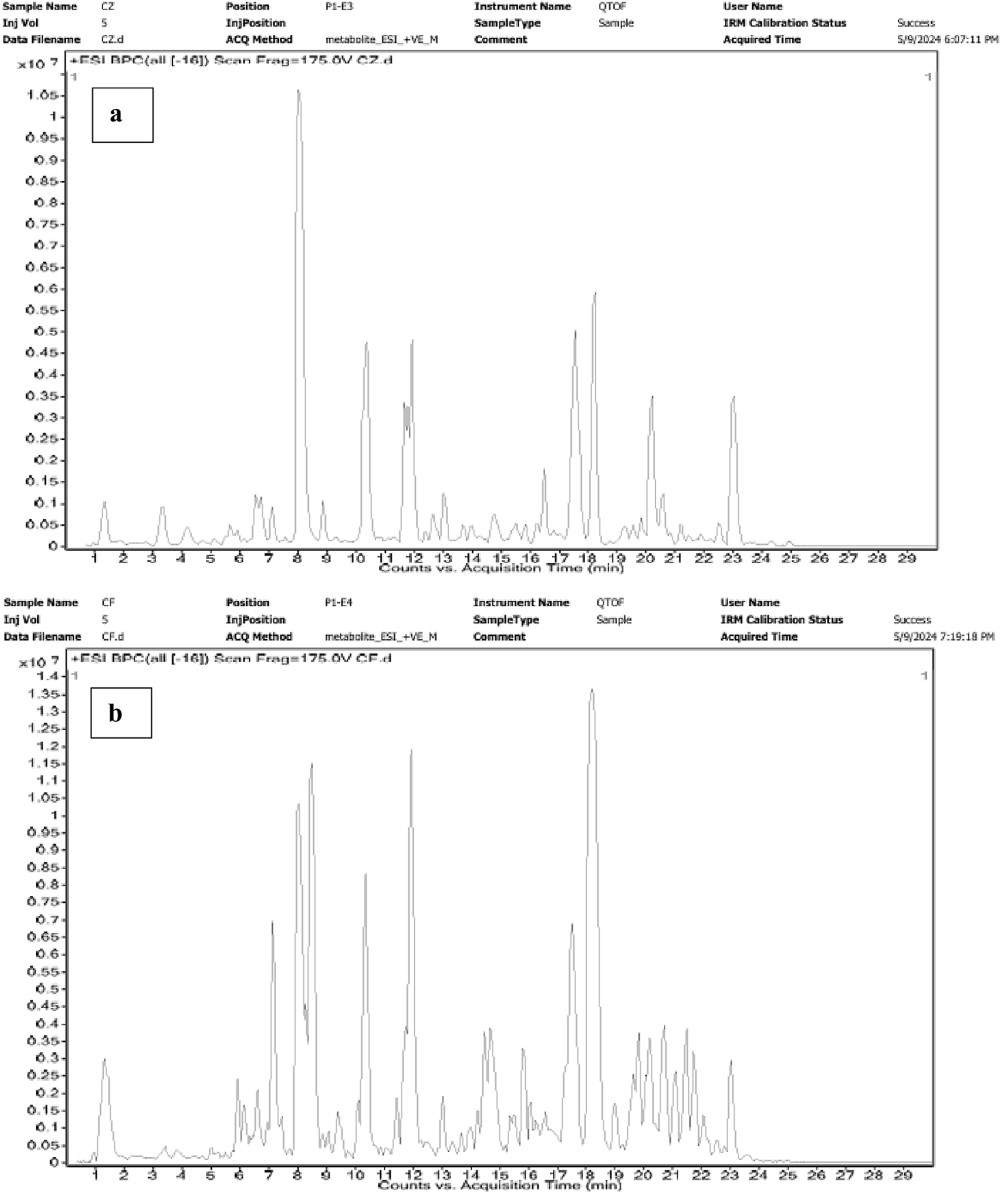
Chromatograms of Z. spinosa **(a)** and F. aegyptia **(b)** chloroform extracts by LC-HRMS method.

In the chloroform extract of *F. aegyptia*, as illustrated in Figure 5b and Table 4, a rich diversity of compounds is observed. This extract contains 17 different compounds with varying area percentages. The major constituents include linolenic acid (12.68%), linolenic acid ethyl ester (7.31%), arachidonic acid (6.96%), (Z)-13-docosenamide (6.47%), and phytol (5.6%). Additionally, the extract features several minor compounds, ranging from cholesterol at 4.64% to 2(4H)-benzofuranone, 5,6,7,7a-tetrahydro-4,4,7a-trimethyl-, (R)- at 0.82%. Common constituents found in the chloroform extracts of both tested plants include the fatty acid arachidonic acid (6.96% in *F. aegyptia* and 3.73% in *Z. spinosa*), the phytosterol stigmasterol (1.84% in *F. aegyptia* and 22.24% in *Z. spinosa*), the benzofuran loliolide (3.70% in *F. aegyptia* and 0.88% in *Z. spinosa*), and the terpene neophytadiene (1.65% in *F. aegyptia* and 1.24% in *Z. spinosa*). Notably, many of these compounds are reported for the first time in these plants. Another qualitative method, LC-HRMS (as shown in Figures 6a,b), was used to detect phytocompounds in these active extracts. We identified 19 compounds in the chloroform extract of *F. aegyptia* and 38 compounds in the chloroform extract of *Z. spinosa* (Table 5). These constituents belong to various chemical classes, including polyols, alkaloids, carboxylic acids, phenolic compounds, lactones, glycosides, terpene glycosides, indoles, N-acylethanolamines, amines, fatty acyls, p-benzoquinones, phenylpropanes, terpenoids, fatty amides, quassinoids, fatty acids, brassinolides, macrolides, cyclic peptides, coumaric acid, brassinosteroids, steroids, triterpenoid saponins, and acetogenins. In the *F. aegyptia* extract, the major compounds include (3S,4S)-3-hydroxytetradecane-1,3,4-tricarboxylic acid (15.87%), 3-tert-butyl-5-methylcatechol (15.31%), 3-methylbutyl 2-methylpropanoate (6.94%), retronecine (6.91%), and palmitic amide (6.91%). For the *Z. spinosa* extract, the predominant compound was palmitic acid (13.31%), followed by (3β,22R,23R,24S)-3,22,23-trihydroxystigmastan-6-one (12.76%), α-licanic acid (9.11%), cyclopassifloside IV (5.42%), and embelin (4.79%). Only one compound was common to both extracts: quercuslactone, which was present at 4.34% in the *F. aegyptia* chloroform extract and 0.59% in the *Z. spinosa* chloroform extract.

**TABLE 5:** Chemical composition in chloroform extracts by LC-HRMS.

Compounds	Class	Retention time	Formula	[M-H]+ *(m/z)*	[M-H]-*(m/z)*	Percentage area %	
						Fa CH	Zs CH
Quinic acid	Polyol	1.183	C7 H12 O6		191.0555		0.59
Retronecine	Alkaloid	1.251	C8 H13 N O2	156.1007		6.91	
L-Malic acid	Carboxylic acids	1.268	C4 H6 O5		133.0145		2.14
2,6-Dihydroxybenzoic acid	Phenolic compound	3.042	C7 H6 O4		153.0199		0.12
Resorcinol	Phenolic compound	3.51	C6 H6 O2		169.0512		0.08
(Cyclohexylmethyl) pyrazine	Alkaloid	3.707	C11 H16 N2	177.1371		2.03	
Chlorogenic acid	Phenolic compound	4.691	C16 H18 O9		353.0869		0.10
Genistic acid	Phenolic compound	4.838	C7 H6 O2		121.0297		2.75
Caffeic acid	Phenolic compound	4.872	C9 H8 O4		179.036		0.11
O-Cresol	Phenolic compound	5.803	C7 H8 O		167.0704		0.23
Vanillin acetate	Phenolic compound	6.517	C10 H10 O4		193.0523		0.46
Quercuslactone a	Lactone	6.557	C9 H16 O2	179.1051		4.34	0.59
Carnegine	Alkaloid	6.653	C13 H19 N O2	222.1471		3.43	
Solanocapsine	Alkaloid	6.726	C27 H46 N2 O2	453.341		1.08	
Isorhamnetin 3-O-[b-D-glucopyranosyl-(1->2)-a-L-rhamnopyranoside]	Glycoside	7.198	C28 H32 O16		623.1652		1.30
(3S,4S)-3-hydroxytetradecane-1,3,4-tricarboxylic acid	Phenolic compound	7.36	C17 H30 O7	347.2059		15.87	
3-tert-Butyl-5-methylcatechol	Phenylpropanes	10.236	C11 H16 O2	181.1213		15.31	
3-Methylbutyl 2-methylpropanoate	Carboxylic acids esters	10.549	C9 H18 O2	181.1209		6.94	
Cyclobrassinone	Indole	10.268	C11 H8 N2 O2 S		231.0243		1.28
Linoleoyl ethanolamide	n-acylethanolamines	11.29	C20 H37 N O2	346.2715		0.96	
Cinnamoside	Terpene glycosides	11.411	C24 H38 O12	541.2223		1.15	
Phytosphingosine	Amines	11.841	C18 H39 N O3	318.2978		1.63	
(1R,2R,4S)-p-Menthane-1,2,8-triol 8-glucoside	Glycoside	11.97	C16 H30 O8	373.1836		1.48	
Lauroyl diethanolamide	Fatty acyls	12.214	C16 H33 N O3	288.2512		2.55	
Embelin	p-benzoquinones	12,45	C17 H26 O4		293.1776		4.79
2,6-Di-tert-butyl-4-ethylphenol	Phenylpropanes	12.907	C16 H26 O	257.1878		1.19	
3-Methyl-alpha-ionyl acetate	Terpenoids	12.954	C16 H26 O2	273.1819		1.04	
Palmitic amide	Fatty amides	13.415	C16 H33 N O		300.2554		0.07
Nigakilactone B	Quassinoids	13.644	C22 H32 O6	415.2095		1.57	
(S)-Nerolidol 3-O-[a-L-Rhamnopyranosyl-(1->4)-a-L-rhamnopyranosyl-(1->2)-b-D-glucopyranoside]	Glycoside	14.768	C33 H56 O14		675.3639		1.28
Gnididilatin	Terpenoid	14.831	C37 H48 O10		711.3424		1.30
Terminaline	Alkaloid	15.003	C23 H41 N O2	364.3184		2.06	
Alpha-licanic acid	Fatty acid	15.515	C18 H28 O3		291.1985		9.11
Homodolicholide	Brassinolides	17.137	C29 H48 O6		491.339		1.67
Ethylene brassylate	Macrolides	17.698	C15 H26 O4	293.1721		1.93	
Pandamine	Cyclic peptide	18.046	C31 H44 N4 O5		549.3326		1.54
16-Feruloyloxypalmitate	Coumaric acids	18.98	C26 H40 O6		447.2776		1.52
Kanokoside D	Terpene glycosides	19.86	C27 H44 O16		623.2565		1.30
Pteroside A	Glycoside	20.11	C21 H30 O8		455.1934		1.29
Gallic acid	Phenolic compound	20.158	C7 H6 O5		169.0143		0.05
19-Hydroxycinnzeylanol 19-glucoside	Terpene glycosides	20.188	C26 H42 O13		607.2593		1.28
Oleamide	Fatty amides	20.443	C18 H35 N O	282.2771		1.04	
N-(14-Methylhexadecanoyl) pyrrolidine	Alkaloid	21.214	C21 H41 N O		368.3186		1.57
Palmitic acid	Fatty acids	21.43	C16 H32 O2		255.2347		13.31
4-Methoxycinnamoyloleanolic acid methyl ester	Terpenoid	21.473	C41 H58 O5		629.4246		1.26
6-Deoxotyphasterol	Brassinosteroid	21.87	C28 H50 O3		479.374		2.00
Teasterone	Steroid	21.885	C28 H48 O4		447.3514		4.31
N-Hexadecanoylpyrrolidine	Alkaloid	22.507	C20 H39 N O	310.3085		2.1	
Cyclopassifloside IV	Triterpenoid saponin	22.965	C37 H62 O12		697.4115		5.42
(3beta,22R,23R,24S)-3,22,23-trihydroxystigmastan-6-one	Steroid	22.97	C29 H50 O4		461.3658		12.76
Annoglaxin	Acetogenins	22.988	C35 H62 O8		669.4559		1.49
3-cis-Hydroxy-b,e-Caroten-3′-one	Terpenoid	23.139	C40 H54 O		605.4016		1.49
Cholesteryl beta-D-glucoside	Glycoside	23.287	C33 H56 O6		607.4235		1.73
Cyclopassifloside II	Triterpene	23.303	C37 H62 O11		681.4183		1.41
Soyasapogenol B 24-O-b-D-glucoside	Triterpenoid saponin	23.349	C36 H60 O8		679.4378		1.35
3,7-Dihydroxy-25-methoxycucurbita-5,23-dien-19-aL	Steroid	23.518	C31 H50 O4		531.371		1.24

### 3.5 Molecular docking study

Molecular docking is a computational method commonly used to obtain insights into the molecular mechanisms of pharmacologically active substances. Several previous studies have highlighted the significant interactions of molecules related to their antibacterial potential through docking simulations (Lahmadi et al., 2023; Horchani et al., 2022; Horchani et al., 2020).

**FIGURE 7:**
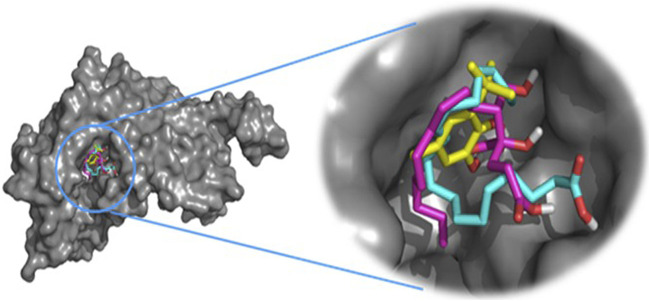
The three-dimensional binding modes of the docked ligands. Linolenic acid (cyan color), (38,48)-3-hydroxytetradecane-1,3,4-tricarboxylic acid (purple color), and 3-tert-Butyl-5-methylcatechol (yellow color)) of the Farsetia aegyptia chloroformic extract in the binding cavity of penicillin-binding protein four (PBP4) from *Enterococcus faecalis* in the ceftaroline-bound form (pdb: 6mki).

**FIGURE 8:**
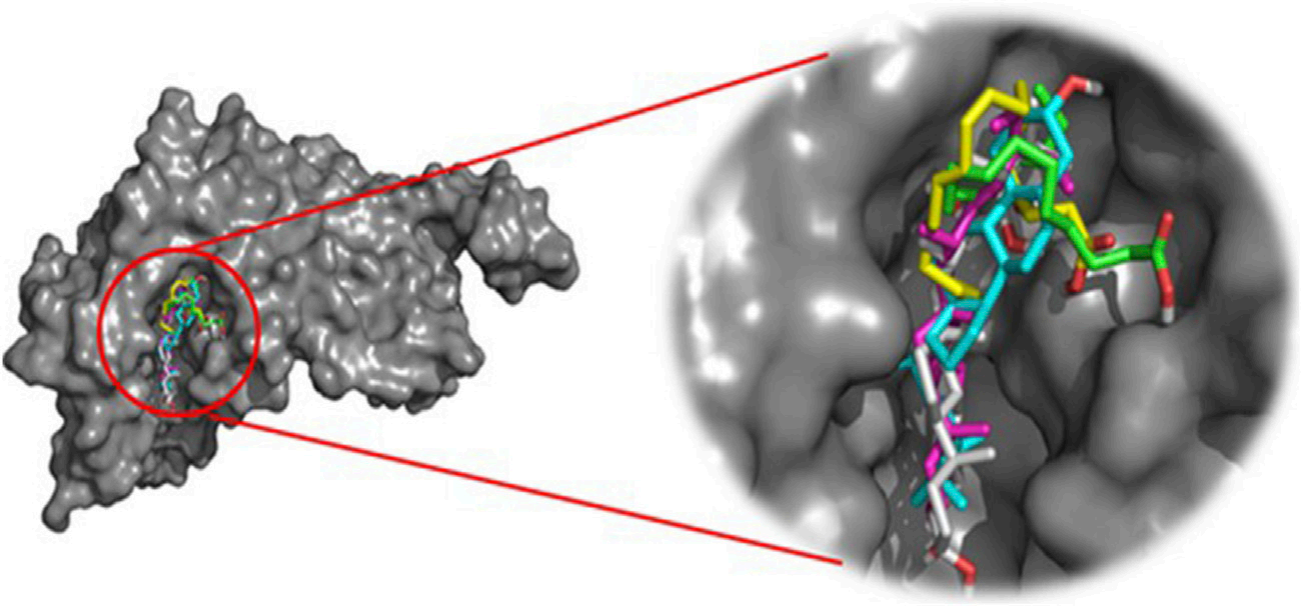
The 3D binding modes of the docked ligands: beta-Sitosterol (cyan color), Stigmasterol (purple color), Alpha-licanic acid (yellow color), Palmitic Acid (green color) and (3beta,22R,23R,248)-3,22,23- Trihydroxystigmastan-6-one (white color)) of the Zilla spinosa chloroformic extract in the binding cavity of penicillin-binding protein four (PBP4) from *Enterococcus faecalis* in the ceftaroline-bound form (pdb: 6 mki).

This approach allows us to identify a ligand (the tested compound) in chloroformic extracts of *F. aegyptia* and *Z. spinosa* that inhibits a specific target protein through its interactions. We selected several targets to predict the effectiveness of the tested ligands in inhibiting the relevant enzymes. According to the results of GC/MS and LC-HRMS, the major compounds found in the chloroformic extract of *Farsetia. aegyptia* include linolenic acid, (3S,4S)-3-hydroxytetradecane-1,3,4-tricarboxylic acid, and 3-tert-butyl-5-methylcatechol. Concerning *Z. spinosa,* the major components of the chloroformic extract are beta-sitosterol, stigmasterol, alpha-linolenic acid, palmitic acid, and (3beta,22R,23R,24S)-3,22,23-trihydroxystigmastan-6-one. These compounds were selected as ligands for docking simulations with the receptor, penicillin-binding protein 4 (PBP4) of *E. faecalis,* in the ceftaroline-bound form (PDB: 6mki), which is implicated in the biosynthesis of peptidoglycan. Both extracts exhibited similar antibacterial potential, with a MIC and MBC of 625 μg/mL against the *E. faecalis*. The *in silico* study aims to identify the compounds responsible for enhancing biological activity. Validation of the docking results for the main abundant compounds from the extracts of the two plants can be observed in Figures 7, 8, which demonstrate that the docked ligands fit well within the target receptor’s active site.

The results presented in Table 6, shown as free-binding energy values, highlight the effects of the selected molecules on the target receptor when compared to the standard, Cefalotin, which has a binding energy of −5.5 kcal/mol. Some of the docked phytocompounds demonstrate higher activity than this standard.

**TABLE 6:** Docking results of the docked ligands with the lowest binding energy score and interacting residues within the binding cavity of penicillin-binding protein four (PBP4) from *Enterococcus faecalis* in the ceftaroline-bound form (pdb:6mki).

Docked molecule	Interacting residues	Binding energy (kcal/mol)
Linolenic acid ^(f)^	Van der Waals: Ser424, Lys427, Thr465, Arg466, Ser482, Asn484, Tyr605, Thr620, Gly621, Thr622, Ser663, Thr665; H bond: Asp666; Alkyl/Pi–Alkyl: Val467	−4.4
(3S,4S)-3-hydroxytetradecane-1,3,4-tricarboxylic acid ^(f)^	Van der Waals: Val467, Ser482, Tyr605, Gly621, Ser637, Phe638, Ser658, Ala664, Asp666; H bond: Thr620, Thr622, Glu635, Ser663, Thr665; carbon hydrogen bond: Thr620	−5.0
3-tert-Butyl-5-methylcatechol ^(f)^	Van der Waals: Gly621, Ser637, Phe638, Ser663, Thr665; H bond: Thr622; Pi-donor hydrogen bond: Thr620; Pi-sigma: Tyr605; Pi-Pi-Stacked: Tyr605; Alkyl/Pi–Alkyl: Val467, Tyr605	−5.1
Beta-sitosterol ^(z)^	Van der Waals: Ser424, Ser482, Asn484, Gln542, Thr620, Gly621, Thr622, Glu624, Ser663, Thr665; H bond: Asp666; Alkyl/Pi–Alkyl: Val467, Tyr540, Tyr605	−7.4
Stigmasterol ^(z)^	Van der Waals: Ser482, Thr620, Gly621, Thr622, Glu624, Ser663, Thr665; H bond: Asp666; Alkyl/Pi–Alkyl: Val467, Tyr605; carbon hydrogen bond: Tyr605	−7.7
Alpha-licanic acid ^(z)^	Van der Waals: Ser424, Val467, Ser482, Tyr605, Thr620, Gly621, Glu635, Ser663; H bond: Thr622	−4.2
Palmitic acid ^(z)^	Van der Waals: Val467, Tyr605, Thr620, Gly621, Thr622, Ser637, Ser658, Gly659, Gly662, Thr665; H bond: Glu635, Ser663	−3.7
(3beta,22R,23R,24S)-3,22,23-trihydroxystigmastan-6-one ^(z)^	Van der Waals: Ser424, Ser482, Asn484, Thr465, Tyr540, Gln542, Gly621, Glu624, Ser637, Ser663, Thr665; H bond: Thr620, Thr622 Pi-sigma: Tyr605; alkyl: Val467	−7.5
Cefalotin (standard)	Van der Waals: Thr465, Arg466, Val467, Ser482, Tyr540, Gln542, Tyr605, Thr620, Gly621; H bond: Thr622, Ser637; carbon hydrogen bond: Ser663; Pi-donor hydrogen bond: Asn484	−5.5

(z): Zilla spinosa; (f): Farsetia aegyptia.

**FIGURE 9:**
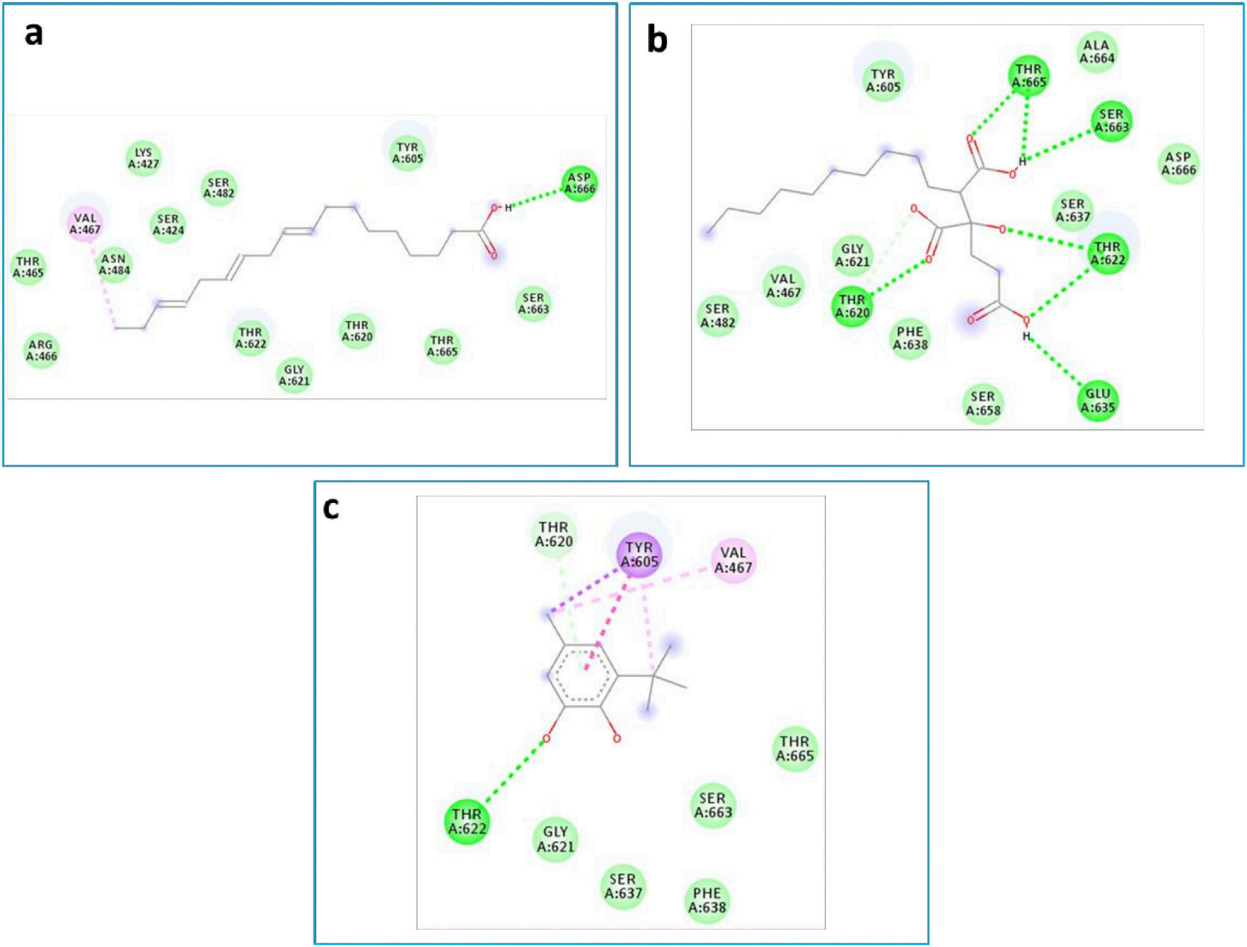
2D docking models of different interactions formed by the docked molecules: **(a)** Linolenic acid, **(b)** (38,4S)-3-hydroxytetradecane-1,3,4-tricarboxylic acid and **(c)**: 3-tert-Butyl-5-methylcatechol of the *F. aegyptia* extract.

**FIGURE 10:**
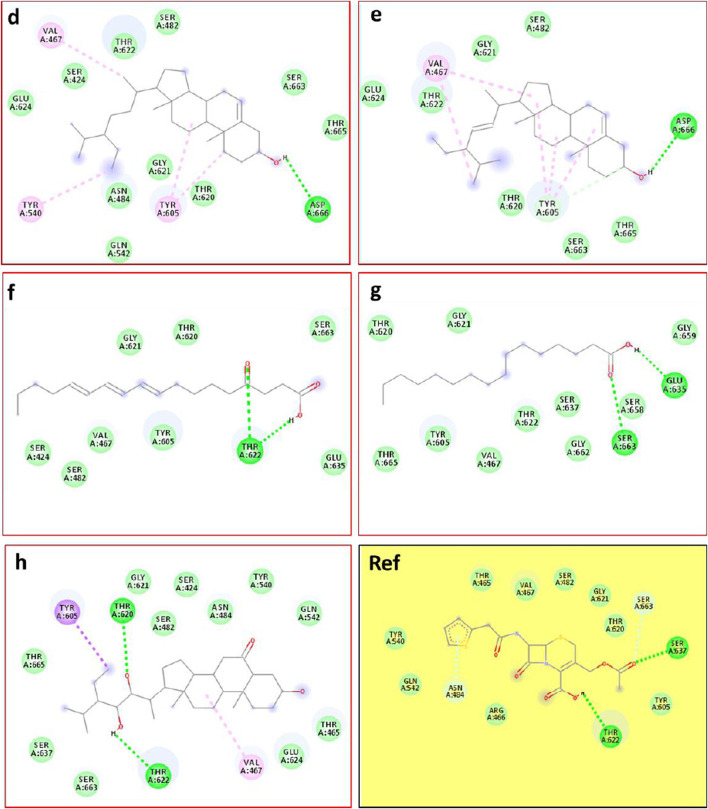
2D docking models of different interactions formed by the docked molecules: **(d)** Beta-sitosterol, **(e)** Stigmasterol, **(f)** Alpha-licanic acid, **(g)** Palmitic acid, **(h)**: (3beta,22R,23R,24S)-3,22,23- trihydroxystigmastan-6-one of the *Z. spinosa* extract and Ref: Cefalotin in the binding cavity of penicillin-binding protein four (PBP4) from *Enterococcus faecalis* in the ceftaroline-bound form (pdb: 6mki).

As is known, the docking is used to predict which compounds within an extract exhibit the most favorable binding scores and are therefore responsible for the biological activity. This biological potential is expressed by inhibiting of the selected target receptor, which is evaluated during the docking process. Such inhibition occurs when the tested molecule forms intermolecular interactions with the amino acids located in the receptor’s active site. Among these interactions, hydrogen bonds are generally the most significant, followed by hydrophobic contacts, including π-related interactions. Thus, molecular interactions such as hydrogen bonds (H-bonds) and π-interactions often correlate strongly with a compound’s the *in vitro* biological activity because they determine how well a ligand binds to its biological target. For example, in our case, the target is “penicillin-binding protein four (PBP4) of *E. faecalis* in the ceftaroline-bound form (PDB: 6mki,” which designates the most sensitive strain according to the *in vitro* test: *E. faecalis* ATCC 29212. In this light, as shown in Figure 9, regarding the *F. aegyptia* extract, we note that 3-tert-Butyl-5-methylcatechol (−5.1 kcal/mol) is the most effective ligand. It forms a hydrogen bond with Thr622 via its hydroxyl group, as well as a Pi-donor hydrogen bond with Thr620, a Pi-sigma bond with Tyr605, and both Pi-Pi stacking and alkyl/Pi-alkyl interactions with Val467 and Tyr605 (Figure 9c). The compound (3S,4S)-3-hydroxytetradecane-1,3,4-tricarboxylic acid (−5 kcal/mol) exhibits similar interactions as follows: hydrogen bonds with Thr620, Thr622, Glu635, Ser663, and Thr665, along with a carbon-hydrogen bond with Thr620. This compound is the second most active among those tested (Figure 9b). In contrast, linolenic acid is the least active ligand when compared to the other phytocompounds from *F. aegyptia*, showing only a hydrogen bond with Asp666 and alkyl/Pi-alkyl contact with Val467 (Figure 9a). On the other hand, concerning the phytoligand from *Z. spinosa*, stigmasterol (−7.7 kcal/mol) has the best docking score. It exhibits a hydrogen bond with Asp666 through its hydroxyl group, as well as alkyl/Pi-alkyl interactions with Val467 and Tyr605. The residue Tyr605 also forms a carbon-hydrogen bond with the carbon skeleton of the molecule (Figure 10e). Additionally, (3beta,22R,23R,24S)-3,22,23-trihydroxystigmastan-6-one, the second-most effective ligand, interacts through two hydrogen bonds with Thr620 and Thr622, a Pi-sigma bond with Tyr605, and an alkyl contact with Val467 (Figure 10h). Beta-sitosterol (−7.4 kcal/mol), like stigmasterol, forms a hydrogen bond with Asp666 via its hydroxyl group, in addition to alkyl/Pi-alkyl interactions with Val467, Tyr540, and Tyr605 (Figure 10d). Meanwhile, the two acids, alpha-licanic acid and palmitic acid, were found to be the weakest docked ligands, displaying some interactions detailed in Table 6 (Figures 10f,g). The above findings demonstrate that the predicted antibacterial effect of the *Z. spinosa* extract against PBP4 from *E. faecalis* in the ceftaroline-bound form primarily results from the three docked compounds: stigmasterol, (3beta,22R,23R,24S)-3,22,23-trihydroxystigmastan-6-one, and beta-sitosterol. Additionally, the individual biological potential of the three major compounds from the *F. aegyptia* extract is lower than that of the compounds from the *Z. spinosa* extract, and even lower than that of the standard. We conclude that the significant antibacterial activity arises from the combination of these three molecules.

The *in silico* molecular docking analysis provided valuable insights into the antibacterial potential of compounds derived from *F. aegyptia* and *Z. spinosa*.

In particular, the phytoconstituents from *Z. spinosa* showed stronger binding affinities and more extensive interactions with the active residues of PBP4 in *E. faecalis* than those from *F. aegyptia*. This result is encouraging for the development of new antibacterial agents. Particularly, stigmasterol (−7.7 kcal/mol), (3β,22R,23R,24S)-3,22,23-trihydroxystigmastan-6-one, and β-sitosterol formed multiple hydrogen bonds, π–σ interactions, and alkyl/π–alkyl contacts with key residues, including Thr620, Thr622, Tyr605, Asp666, and Val467. This accentuates their potent stabilizing potential at the active site. On the other hand, the most active phytoconstituents from *F. aegyptia*, namely, 3-tert-butyl-5-methylcatechol and (3S,4S)-3-hydroxytetradecane-1,3,4-tricarboxylic acid, displayed weaker binding energies (−5.0 to −5.1 kcal/mol) and fewer stabilizing interactions. In addition, palmitic acid and linolenic acid, which are found in both species, were estimated to be the least efficient ligands. These results are consistent with earlier publications that emphasize the antimicrobial activity of phytosterols, mainly those found in *Z. spinosa* (Kanokmedhakul et al., 2005), aligning well with our docking findings, which identified sterols as the most effective ligands. Altogether, the docking study findings suggest that the important antibacterial activity of *Z. spinosa* derives from a synergistic effect of its three active sterols, although the inferior activity of *F. aegyptia* may be linked to its lower levels of interactive fatty acids and phenolic compounds.

## 4 Conclusion

This is the first report of advanced bactericidal activities of the organic extracts of *F. aegyptia* and *Z. spinosa* from Ha’il, Saudi Arabia. Furthermore, it demonstrated antibiofilm activity against Gram-positive, Gram-negative bacterial strains, and anti-candida strains. The molecular docking showed that the major components of *Zilla spinose* have the highest binding free energy with penicillin-binding protein 4 (PBP4) of *E. faecalis* in the ceftaroline-bound form (PDB: 6mki), which is implicated in the biosynthesis and survival of this bacterium. Further *in vivo* studies will be needed to elucidate the specific mechanisms of action and the safety of these important phytochemical components before translating their therapeutic potential.

## Data Availability

The original contributions presented in the study are included in the article/supplementary material, further inquiries can be directed to the corresponding author.
